# Glycooligomer-Functionalized Catalytic Nanocompartments Co-Loaded with Enzymes Support Parallel Reactions and Promote Cell Internalization

**DOI:** 10.1021/acs.biomac.4c00526

**Published:** 2024-06-24

**Authors:** Maria Korpidou, Jonas Becker, Shabnam Tarvirdipour, Ionel Adrian Dinu, C. Remzi Becer, Cornelia G. Palivan

**Affiliations:** † Department of Chemistry, University of Basel, Mattenstrasse 22, Basel 4002, Switzerland; ‡ Department of Chemistry, University of Warwick, Coventry CV4 7AL, United Kingdom; § NCCR Molecular Systems Engineering, Mattenstrasse 22, Basel 4002, Switzerland

## Abstract

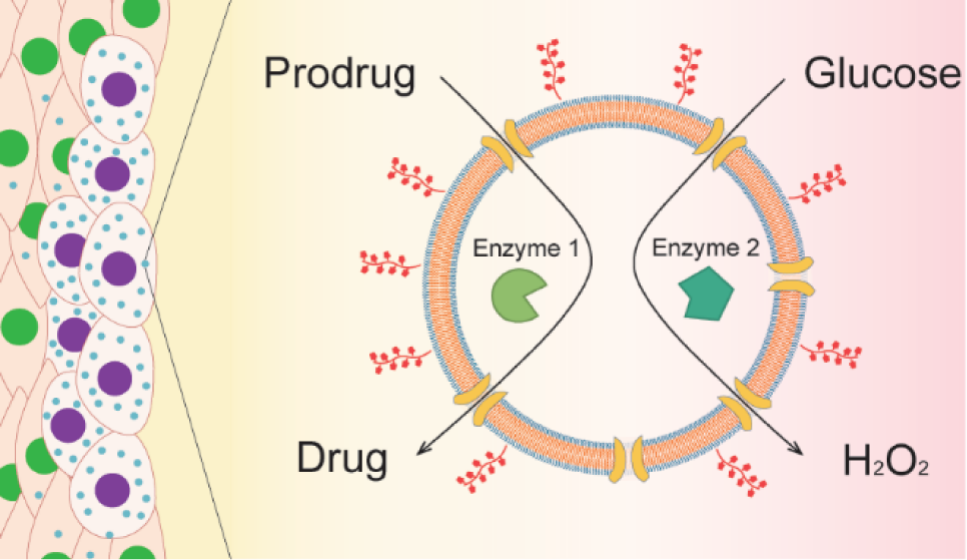

A major shortcoming associated with the application of enzymes in drug synergism originates from the lack of site-specific, multifunctional nanomedicine. This study introduces catalytic nanocompartments (CNCs) made of a mixture of PDMS-*b*-PMOXA diblock copolymers, decorated with glycooligomer tethers comprising eight mannose-containing repeating units and coencapsulating two enzymes, providing multifunctionality by their in situ parallel reactions. Beta-glucuronidase (GUS) serves for local reactivation of the drug hymecromone, while glucose oxidase (GOx) induces cell starvation through glucose depletion and generation of the cytotoxic H_2_O_2_. The insertion of the pore-forming peptide, melittin, facilitates diffusion of substrates and products through the membranes. Increased cell-specific internalization of the CNCs results in a substantial decrease in HepG2 cell viability after 24 h, attributed to simultaneous production of hymecromone and H_2_O_2_. Such parallel enzymatic reactions taking place in nanocompartments pave the way to achieve efficient combinatorial cancer therapy by enabling localized drug production along with reactive oxygen species (ROS) elevation.

## Introduction

Enzyme-based treatments emerge as an innovative therapeutic strategy for a number of pathologies, spanning from metabolic and ocular disorders to cancer.^1,2^ Despite their potential, approval and market release are limited by issues associated with the clinical administration of enzymes, lack of tissue specificity, and potential immunogenicity.^2,3^ Efforts to overcome these hurdles focus on the development of polymer-based nanocompartments for intracellular delivery of enzymes, as they exhibit enhanced colloidal stability and versatile chemistry, supporting fine-tuning of properties and external functionalization.^1,4−7^ One of the main strategies used in the development of enzyme-based therapeutics include their loading inside CNCs, where the enzymes perform their activity in situ.^2,4^ The approach of CNCs has the advantages of protecting the encapsulated enzymes from proteolytic attack,^8^ promoting their efficacy by localizing them in specific bioregions,^6^ and potentially increasing their activity by confinement.^9^ CNCs encapsulating single enzymes have been developed for conversion of prodrugs into their active therapeutic form^10−13^ and detoxification of harmful reactive oxygen species,^9^ or as artificial organelles.^8,14^ Furthermore, single enzyme-containing CNCs exhibiting a dual functionality were introduced,^12,15−17^ following the trends of combinatorial strategies in treating complex pathological conditions, including cancer and addressing multiple facets of the disease.^18,19^ For example, singly loaded β-galactosidase inside polyethylene glycol-*b*-2-(piperidin-1-yl)ethyl methacrylate-*co*-butyl methacrylate polymersomes activated 5-*N*-(β-d- galactopyranosiylbenzyloxy-carbonyl)-doxorubicin (DoxGal) and 1-cyclo-hexyl-2-(5H-imidazo[5,1-*a*]isoindol-5-yl)ethanol, resulting in simultaneous production of doxorubicin and NLG919.^12^ It is noteworthy that achieving cytotoxicity in 4T1 cells comparable to the free form drugs, required an enzyme concentration of 12.5 U mL^–1^. This concentration could be significantly reduced by utilizing compartments that coencapsulate different enzymes capable of simultaneously producing distinct therapeutic molecules and enhancing CNC multifunctionality. While previous studies reported different coencapsulated enzymes inside CNCs for cascade reactions (the product of one enzymatic reaction serves as the substrate for the second), the coencapsulation for parallel reactions remains unexplored.^20,21^ An example of cascade reaction is the superoxide dismutase (SOD)–lactoperoxidase (LPO) pair: SOD catalyzes superoxide radicals to H_2_O_2_, which LPO then converts to water and oxygen.^20^ Despite the advantage of simultaneously producing distinct therapeutic molecules, coencapsulation of different types of enzymes in CNCs for parallel reactions offers a cost-effective and simplified development process to achieve a similar therapeutic response. Meanwhile, nonspecific or insufficient cell uptake of nanosystems remains a challenge.^22^ Incorporation of targeting moieties on the surface of nanocarriers for site-specific delivery and optimal therapeutic response has been largely developed for enzyme delivery carriers^23^ and reported only for a few single-enzyme CNCs acting as advanced artificial organelles.^24,25^ While expected to have significant advantages in terms of efficacy, combinatorial response, and improved up take, integration of multifunctionality and targeting in one nanocarrier still remains sparsely investigated due to the complexity of accommodating both the biofunctionality and spatial localization.

**Figure 1:**
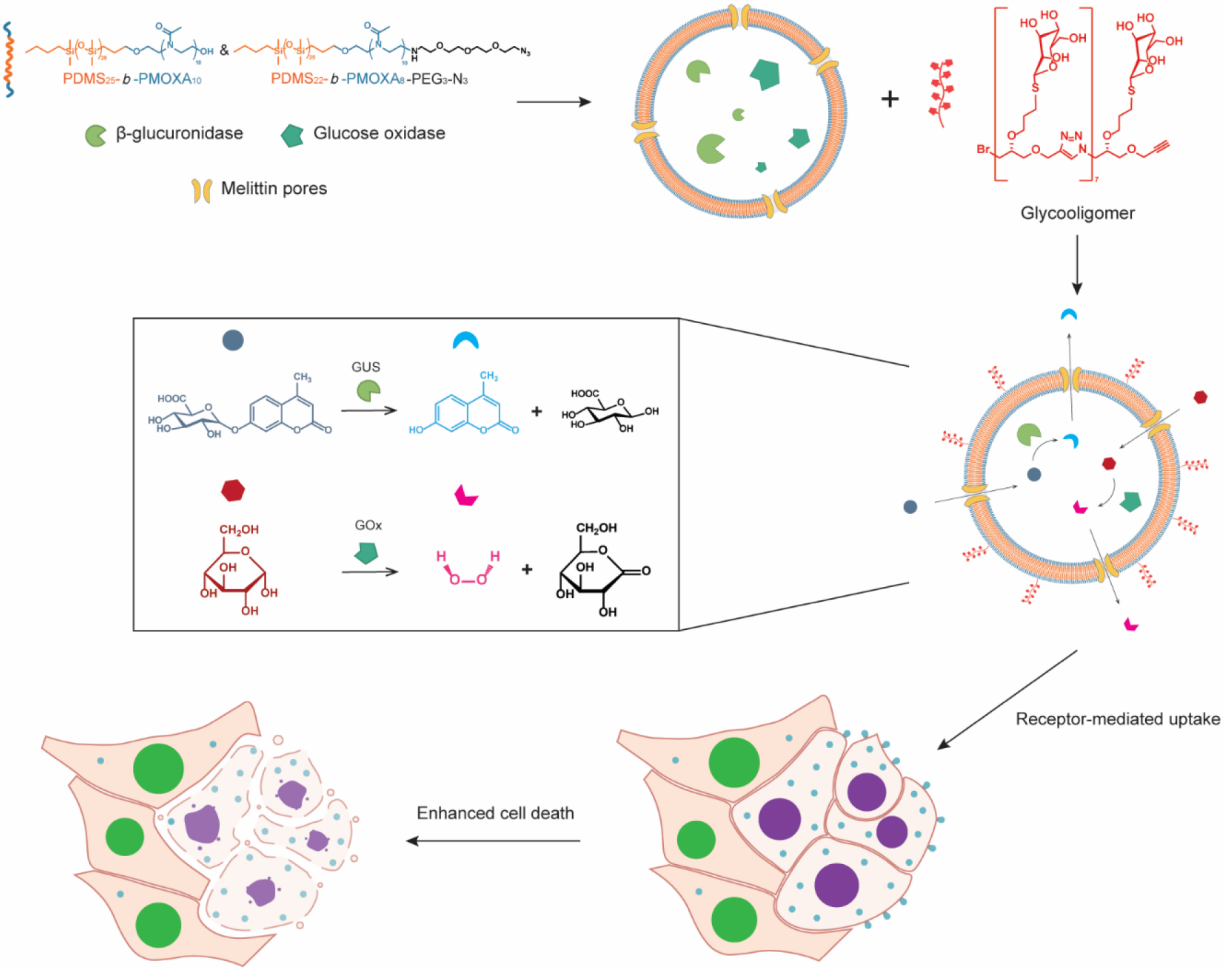
Schematic representation of the formation of glycooligomer-functionalized catalytic nanocompartments (GUS-GOx-CNCs-Gly) and their enzymatic activity. Βeta-glucuronidase (GUS) and glucose oxidase (GOx) are coencapsulated in PDMS-*b*-PMOXA-based nanocompartments decorated with glycooligomer tethers for targeting liver cancer cells. The melittin pores inserted into the polymer membrane facilitate the diffusion of substrates (4-MUG, glucose) and products (4-MU, H_2_O_2_) of the enzymes. Simultaneous production of 4-MU and H_2_O_2_ leads to enhanced cell death.

In our study, we address these challenges by introducing dual enzyme-loaded polymer nanocompartments for catalysis of parallel reactions and they have improved cellular uptake due to decoration with specific targeting molecules. We selected to encapsulate inside polymersomes two enzymes with high potential in combinatorial cancer therapy: GUS, which produces the drug hymecromone (or 4-MU) from its glucuronide conjugate (4-MUG) and GOx, which generates cytotoxic H_2_O_2_ (Figure 1). GUS is an important enzyme in prodrug therapy, cancer prognosis, and hepatoprotection,^26^ while GOx induces cancer cell starvation by consuming glucose in the tumor microenvironment and generates cytotoxic H_2_O_2_.^27,28^ While, within the field of multienzyme therapy, GOx has been paired with enzymes such as catalase and horseradish peroxidase (HRP) for cascade detoxification of the generated H_2_O_2_,^20,29^ it has not yet been explored in parallel reactions to produce a synergistic effect. In particular, to the best of our knowledge, GOx and GUS have not been evaluated in combination so far, in spite of their therapeutic potential. Furthermore, compared to their administration in different compartments,^13,16^ the advantages of coencapsulation would include simplified coadministration and assured local presence of the two enzymes. As nanocompartments, we self-assembled polymersomes from a mixture of unfunctionalized and azide-functionalized poly(dimethylsiloxane)-*b*-poly(2-methyl-2-oxazoline) (PDMS-*b*-PMOXA) diblock copolymers which have been reported to allow the encapsulation of biomolecules in their inner cavity and support attachment of targeting moieties.^13,30,31^ In order for both enzymes to act in situ inside the nanocompartments, a molecular flow through the polymersomes’ membrane is essential. To permeabilize the membrane of the nanocompartments, which is otherwise impermeable,^32^ we selected to insert melittin, a peptide shown to form pores in the membranes of PDMS-*b*-PMOXA or PMOXA-*b*-PDMS-*b*-PMOXA polymersomes.^13,33^

To favor the efficient cellular uptake of the resulting CNCs, glycooligomer tethers consisting of eight pendant mannose units were attached to the azide-groups exposed on the external interface of their membrane. This modification aims to achieve active targeting of mannose-binding receptors (MBRs).^34,35^ MBRs belong to the group of carbohydrate-binding proteins (aka lectins) that provide selective carbohydrate recognition and subsequent endocytosis.^36−40^ MBRs have become the focal point in novel therapeutic approaches as they are highly expressed on the surface of cancer cells and have been associated with the progression of hepatocellular carcinoma (HCC), the sixth most common cancer.^41,42^ As a result, glycooligomers, renowned for their excellent lectin-binding ability are ideal candidates for cell-targeting nanomedicinal systems in cancer therapy.^34,43−48^ Although polymersomes have been functionalized with sugars such as, glucose, galactose, and mannose, glycooligomers are yet to be used.^49,50^ As a proof of concept for cell targeting, we chose two cell lines, each possessing different MBR expression profiles; liver-derived HepG2 cells with high expression levels and cervix-derived HeLa cells expressing lower levels of such proteins.^34,41,51−53^ We explored for the first time the synergistic effect of the in situ produced hymecromone and H_2_O_2_ on the cell viability. Dual enzyme-loaded catalytic nanocompartments for multifunctional response to pathologic conditions and with an improved internalization by efficient targeting opens new avenues in combinatorial treatment of complex pathologic conditions.

## Experimental Section

### Materials

2,2-Dimethoxy-2-phenylacetophenone (99%), sodium methoxide solution (25 wt % in MeOH), tetrabutylammonium fluoride solution (1 M in THF), β-glucuronidase (GUS, *E. coli* Type VII-A), glucose oxidase (GOx, *A. niger* Type VII), peroxidase from horseradish (HRP, Type VI), melittin (from honey bee venom), concanavalin A (FITC-ConA, *C. ensiformis* FITC conjugate, Type IV), 4-methylumbelliferyl-β-d-glucuronide (4-MUG), proteinase K (from *Tritirachium album*), fluorescent dye Atto647, penicillin, streptomycin, l-glutamine, Sepharose (4B, 45–165 μm beads diameter), Whatman Nucleopore Track-Etched membranes (100 nm), (+)-sodium l-ascorbate, Copper(II) sulfate pentahydrate and the Copper Assay Kit were purchased from Sigma-Aldrich (USA). Acetonitrile (MeCN, 99.9%, extra dry) and tetrahydrofuran (99.5%, extra dry) were purchased from Acros Organics (Belgium). Dialysis tubing (Spectrum Spectra Por 7 RC, MWCO 1 kDa) was purchased from Spectrum Laboratories Inc. (USA). 2,3,4,6-*Tetra*-*O*-acetyl-1-thio-β-d-mannopyranose (Ac4ManSH) was synthesized according to known literature procedures.^54^ Atto488 *N*-hydroxysuccinimide ester (Atto488 NHS-Ester) and Atto633 *N*-hydroxysuccinimide ester (Atto633 NHS-Ester) were purchased from ATTO-TEC (Germany). CM5 sensor chips were purchased from Cytiva Life Sciences (USA). MBL, dectin-1, DC-SIGN, and mannose receptor 1 were purchased from Bio-Techne (USA). Enhanced Pierce bicinchonic acid (BCA) assay, calcein, Alexa Fluor 647 alkyne, Amplex Red Reagent, and wheat germ agglutinin–Alexa Fluor 555 conjugate were purchased from Thermo Fisher Scientific (USA). Phosphate-buffered saline (PBS) and fetal bovine serum (FBS) were purchased from BioConcept (Switzerland). Dulbecco′s modified Eagle′s medium (DMEM) and nonessential amino acids (NEAA) were purchased from Gibco Life Sciences (USA). CellTiter 96 AQueous One Solution Cell Proliferation Assay (MTS) and Hoechst 33342 trihydrochloride trihydrate were purchased from Invitrogen (USA). The Reactive Oxygen Species (ROS) Detection Assay Kit (ab287839) was purchased from Abcam (UK). HeLa-GFP (HeLa S3 cells stably expressing histone H2B-GFP) were obtained from the Nigg laboratory (Biozentrum, Basel).^55^ All moisture-sensitive reactions were carried out under an inert atmosphere of nitrogen using standard syringe/septa techniques.

### Nuclear Magnetic Resonance (NMR) Spectroscopy

^1^H NMR spectra of the PDMS-*b*-PMOXA copolymers were recorded at 295 K in a variety of solvents on a Bruker Avance III NMR spectrometer (500 MHz). The instrument was equipped with a direct observe 5 mm BBFO smart probe. Each sample was measured with the default number of 16 scans. All spectra were processed with MestReNova software, and chemical shifts were reported in ppm.

^1^H NMR spectroscopy was also performed for the glycooligomer on a Bruker Avance III HD 400 MHz instrument. Deuterated solvents were used, and the signal of the residual solvent served as a reference for the chemical shift, δ.

### Nanocompartment Preparation

Synthesis and characterization of amphiphilic diblock copolymers poly(dimethylsiloxane)_25_-*block*-poly(2-methyl-2-oxazoline)_10_ (PDMS_25_-*b*-PMOXA_10_) and azide-functionalized poly(dimethylsiloxane)_22_-*block*-poly(2-methyloxazoline)_8_ (PDMS_22_-*b*-PMOXA_8_-OEG_3_-N_3_) were described previously.^30,31^

All nanocompartments in this study were prepared with the film rehydration method.^13,56^ For the formation of the enzyme-containing catalytic nanocompartments equipped with melittin pores and functionalized with the glycooligomer (GUS-GOx-CNCs-Gly), a thin film of PDMS_25_-*b*-PMOXA_10_ and PDMS_22_-*b*-PMOXA_8_-OEG_3_-N_3_ (1:1 mol %, 5 or 10 mg mL^–1^ polymer in EtOH) was formed by rotary evaporation of the solvent (100 rpm at 40 °C, 160 mbar for 45 min). The film was rehydrated by stirring overnight at room temperature (RT) using a solution of PBS containing β-glucuronidase (0.5 mg mL^–1^, 25 kU), glucose oxidase (0.5 mg mL^–1^, 10 kU), and melittin (50 μM). Control nanocompartments lacking any of the components were prepared according to the same procedure, but missing the corresponding molecules. For Cy5-melittin polymersomes, the film was rehydrated with PBS containing Cy5-melittin (25, 50, or 75 μM). For Atto647-encapsulating polymersomes, the film was rehydrated with PBS containing Atto647 (0.02 mg mL^–1^). To remove nonencapsulated proteins, nanocompartments were incubated with proteinase K (0.05 mg mL^–1^) for 2 h at 37 °C, following extrusion (21 times) through a 100 nm Whatman Nuclepore polycarbonate membrane. For further purification, size exclusion chromatography (SEC, Sepharose 4B, PBS) was performed. CuAAC was performed for the functionalization of the outer polymer membrane with the glycooligomer. Nanocompartments were incubated with the glycooligomer (1:1 mol ratio of azide-terminated polymer and glycooligomer, PBS,(+)-sodiuml-ascorbate 10 mol %, copper(II) sulfate pentahydrate 1 mol %) overnight and stirring at RT. Size exclusion chromatography (Sepharose 4B, PBS) was performed for further purification. Nanocompartment suspensions were stored at 4 °C until further use.

## Characterization of Nanocompartments

### Light Scattering

Dynamic light scattering (DLS) experiments were performed using a Zetasizer Nano ZSP (Malvern Instruments Ltd., U.K.) (0.3 mg mL^–1^ polymer, RT, λ = 633 nm, 173°). Measurements were carried out in triplicate, 11 runs each.

Static light scattering (SLS) experiments were performed on a light scattering spectrometer (LS instruments, Switzerland) (0.03 mg mL^–1^ polymer, 25 °C, He–Ne 21 mW laser, λ = 632.8 nm, 30° to 135°). The radius of gyration (*R*_g_) was obtained from the SLS data using Guinier plots, while the hydrodynamic radius (*R*_h_) was obtained from DLS.

### Zeta Potential

Zeta potential was measured in a Zetasizer Nano ZSP (Malvern Instruments Ltd., U.K.). Samples Diluted in water were placed in a disposable folding capillary DTS1070 cuvette, and the zeta-potential was recorded after each polyelectrolyte deposition.

### Nanoparticle Tracking Analysis (NTA)

Nanoparticle tracking analysis (NTA) was performed using a NanoSight NS 300 instrument (NanoSight Ltd., U.K.) (0.0125 mg mL^–1^ polymer, 25 °C, λ = 532 nm). A measurement consisted of a 60 s video and performed in triplicate. The mean and median size, and the concentration of the nanocompartments in the solution were obtained using the NTA software (version 3.4, NanoSight).

### Transmission Electron Microscopy (TEM)

Nanocompartments (5 μL, 0.2 mg mL^–1^ polymer) were adsorbed on 400 mesh copper grids (1 min), washed with water and negatively stained (uranyl acetate (2%), 10 s). Samples were washed and blotted, and transmission electron microscopy micrographs were recorded on a Philips CM100 with an accelerating voltage of 80 kV.

### Fluorescence Correlation/Cross-Correlation Spectroscopy (FCS/FCCS)

GUS and GOx were labeled with Atto488 NHS-Ester and Atto633 NHS-Ester, respectively. Briefly, the enzymes were incubated with a 7-fold excess of the dye (4 °C, dark, stirring, 48 h) and the unconjugated dye was removed by SEC (Sepharose 4B, PBS).

For fluorescence correlation/cross-correlation spectroscopy (FCS/FCCS), an inverted laser scanning confocal microscope (LSM 880, Carl Zeiss, Germany) with a water immersion objective (Zeiss C/Apochromat, *M* = 40, NA = 1.2) was used. Atto488 was excited with an argon laser (λ = 488 nm, MBS 488 filter), and Atto633 and Cy5 were excited using a HeNe laser (λ = 633 nm, MBS 488/561/633 filter). The pinhole size (1 AU) was adjusted before recording autocorrelation curves of the free fluorophores.

For FCS measurements, free fluorophores, fluorophore labeled-enzymes, or nanocompartments in PBS (20 μL), were placed on a 0.15 mm thick glass coverslip. Fluorescent fluctuations from free fluorophores, labeled enzymes, and nanocompartments loaded with labeled enzymes and Cy-5 melittin nancompartments were measured over time. Fluorescence cross-correlation spectroscopy (FCCS) was performed similarly to FCS on nanocompartments containing both of the labeled enzymes, in FCCS mode. The ZEN software was used for the processing and analysis of the raw data. Eq 1, i.e., the one component diffusion model was used for fitting the experimental autocorrelation curves for the free fluorophores:
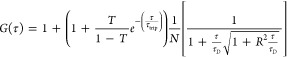
1where *N* is the average number of particles in the observation volume, τ_D_ is the diffusional correlation time, and *R* is the structural parameter (5). *T* is the fraction of molecules in triple state, and τ_trip_ is the triplet time. Using the relationship between the x–y dimension of the confocal volume (ω_*xy*_) and τ_D_, the diffusion coefficient *D* was calculated as in eq 2:
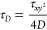
2

Eq 3 represents the two-component diffusion model used for fitting the experimental autocorrelation curves for the free labeled enzymes, the nanocompartments encapsulating labeled enzymes and the Cy-5 melittin nancompartments:
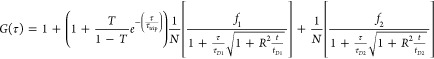
3

The number of dye molecules per enzyme (NPE) was calculated using eq 4, and the number of enzymes per nanocompartment (NPN) using Eq 5:
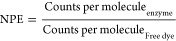
4
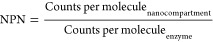
5

The number of melittin pores per nanocompartment (NMP) was calculated using eq 6:
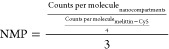
6

The hydrodynamic radius (*R*_h_) of the nanocompartments was calculated using Einstein–Stokes eq 7, where *D* is the diffusion coefficient, *k*_B_ – Boltzmann’s constant, *T* – absolute temperature, and η – viscosity of the surrounding medium.
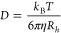
7

### Concanavalin a (ConA) Clustering Assay

Glycooligomer-functionalized Atto647-encapsulating polymersomes or nonfunctionalized polymerosmes (PBS, 0.85 mg mL^–1^) were incubated (1 h, RT) with FITC-ConA (PBS, 0.65 mg mL^–1^). Samples (20 μL) were placed on a 0.15 mm thick glass coverslip and imaged on an inverted laser scanning confocal microscope (LSM 880, Carl Zeiss, Germany) with a water immersion objective (Zeiss C/Apochromat, *M* = 40, NA = 1.2). FITC was excited using an argon laser (λ = 488 nm, MBS 488 filter), and Atto647 using a HeNe laser (λ = 633 nm, MBS488/561/633 filter). The ImageJ software was used to analyze the images and calculate the Pearson’s colocalization coefficient.

### Surface Plasmon Resonance (SPR)

Surface plasmon resonance (SPR) was used to determine the extent of interaction between the glycooligomer-functionalized polymersomes and lectins. Samples were analyzed on a BIAcore T200 system (Cytiva Life Sciences). The lectins (25 μg mL^–1^) were immobilized via a standard amino coupling protocol onto a CM5 sensor chip that was activated by flowing a 1:1 mixture of 0.1 M *N*-hydroxysuccinimide (NHS) and 0.4 M *N*-ethyl-*N*’(dimethylaminopropyl)-carbodiimide (EDC) over the chip for 5 min at 20 °C at a flow rate of 5 μL min^–1^ after system equilibration with HEPES-buffered saline (HBS) buffer (10 mM HEPES pH 7.4, 150 mM NaCl, 5 mM CaCl_2_). Subsequently, channels 1 (blank), 2, 3, and 4 were blocked by flowing a solution of ethanolamine (1 M pH 8.5) for 10 min at 5 μL min^–1^ to block the remaining reactive groups on the channels. Sample solutions were prepared at varying concentrations (100–3.125 μM) in the same HBS buffer to calculate the binding kinetics. Sensorgrams for each sample concentration were recorded at 20 °C with a flow rate of 25 μL min^–1^. Injection of polymer solution 350 s (on period) was followed by 200 s of buffer alone (off period). Regeneration of the sensor chip surfaces was performed using a solution of 10 mM HEPES pH 7.4, 150 mM NaCl, 10 mM EDTA, 0.01% Tween 20. All binding curves were subjected to double referencing by subtracting the signal of a reference channel without protein on the chip and the signal of a blank buffer injection. Kinetic data was evaluated using a 1:1 Langmuir binding model in the BIA evaluation 3.1 software.

### Estimation of Glycooligomers per Nanocompartment

The number of glycooligomers per nanocompartment (GPN) was calculated using eq 8:
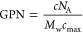
8

where *c* is the concentration of glycooligomer in the total volume of polymersomes, *N*_A_ is the Avogadro number, *M*_w_ is the molecular weight of the glycooligomer, and *c*_max_ is the maximum concentration of polymersomes. For the estimation, the absorbance of glycooligomer (0.05 mg mL^–1^), empty polymersomes (0.1 mg mL^–1^ polymer), and glycooligomer-functionalized polymersomes (0.1 mg mL^–1^ polymer) was measured at λ = 250 nm on a Nanodrop 2000c UV–vis spectrophotometer (ThermoFisher, USA). The absorbance value of empty polymersomes was subtracted from the glycooligomer-functionalized polymersomes’ value and used for the calculation of glycooligomer concentration in the sample. The concentration of polymersomes was determined by NTA using a NanoSight NS300 device (Malvern, U.K.).

### BCA Assay for Determining Enzyme Encapsulation Efficiency

In the case of unlabeled enzymes, the amount of encapsulated proteins was calculated by subtracting the amount of protein in CNCs from the initial, total amount of protein used in the film rehydration solution. The quantification of protein was conducted by the enhanced Pierce bicinchonic acid (BCA) assay according to the supplier’s protocol with the following modifications; a calibration curve was prepared with different concentrations of GUS and GOx. Nonpermeabilized, nonfunctionalized GUS-GOx–CNCs were first ruptured by sonication and then incubated with ethanol at a ratio of 3:1 (v/v) for 1 h at 37 °C. The solution was added at a 1:2 ratio to the BCA reagent. Samples and standards were incubated for 1 h at 37 °C, and the absorbance was measured at 562 nm using a SpectraMax id3 plate reader (Molecular Devices, USA).

### Detection of Copper

The Copper Detection Kit was used according to the manufacturer’s protocol with the following modifications. Cu^2+^ standards (0, 15, 30, and 45 μM) and samples were prepared by adding Reagent A (3:1 v/v) and mixing well, and transferred to a 96-well plate. Master Reaction Mix was prepared by adding Reagent B and Reagent C in a 1:30 (v/v) ratio, added to the wells in a 1.5:1 ratio (v/v) to the samples and standards and mixed well. The plate was incubated at RT for 5 min in dark, and absorbance was measured at 359 nm using a SpectraMax id3 plate reader (Molecular Devices, USA). A calibration curve was constructed for estimating the amount of Cu^2+^ in the samples.

### Enzyme Activity Assays

Fluorescence assays were performed using 96-well, flat bottom black plates (Thermo Fisher Scientific). The increase of fluorescence for 4-MU (λ_ex_: 365 nm, λ_em_: 445 nm) and resorufin (λ_ex_: 550 nm, λ_em_: 590 nm) was measured in a SpectraMax id3 microplate reader (Molecular Devices, USA). 4-Methylumbelliferyl-β-d-glucuronide (4-MUG, 10 μM) was added to CNCs ([GUS] 13 μg mL^–1^, [GOx] 97 μg mL^–1^) or the respective amount of free enzymes in solution in PBS or PBS containing 50% Dulbecco’s modified Eagle medium (DMEM) Phenol Red free with 10% fetal bovine serum ([glucose] 12.5 mM) per well (100 μL). 4-MUG (10 μM) without CNCs or free enzymes was added in the mixture as a control. Fluorescence emission (λ_em_: 445 nm) was monitored for 60 min at 37 °C. For evaluating the production of H_2_O_2_, reactions were then diluted 1:1250, and HRP ([HRP] 100 μg mL^–1^) and Amplex Red ([Amplex Red] 10 μM) were added. Fluorescence emission (λ_em_: 590 nm) was monitored for a total of 2 h at 37 °C.

For estimating 4-MU and resorufin production, the fluorescence intensity of 0, 2, 4, 6, 8, and 10 μM 4-MU and 0, 1.5, 3, 4.5, 6, and 7.5 μM resorufin in PBS containing 50% Dulbecco’s modified Eagle medium (DMEM) Phenol Red free with 10% fetal bovine serum was measured, and calibration curves were constructed. 4-MU and resorufin produced by free and encapsulated enzymes was calculated based on their calibration curves when the reaction reached maximum fluorescence intensity.

### Cell Cultures

HepG2 (hepatocellular carcinoma, human; ATCC HB-8065) and HeLa S3–H2B-GFP (cervical adenocarcinoma, human) cells were routinely cultured in DMEM supplemented with 10% FBS, 100 U mL^–1^ penicillin, 100 U mL^–1^ streptomycin, 1% l-glutamine, and 1% nonessential amino acids. Cells were maintained at 37 °C under a humidified atmosphere containing 5% CO_2_.

### Cellular Uptake and Imaging

Cells (HepG2 or HepG2 and HeLa S3 H2B-GFP) were seeded at a concentration of 30 000 cells (300 μL) in each well of an ibidi 8-well chambered glass bottom coverslip (Vitaris, Switzerland). After 24 h, the medium was replaced with fresh DMEM containing either glycooligomer-functionalized Atto647-encapsulating polymersomes (0.3 mg mL^–1^) or nonfunctionalized polymersomes (0.3 mg mL^–1^). Following a 24-h incubation, cells were washed with PBS (3×), fixed with 4% paraformaldehyde (15 min, RT), and rinsed with PBS (3×). Nuclei were stained by incubating cells with a 20 000-fold dilution of Hoechst 33342 fluorescent dye (20 min, 37 °C), followed by washing (PBS, 3×). To stain their membrane of cocultured cells, cells were incubated with the wheat germ agglutinin–Alexa Fluor555 conjugate (200-fold dilution, 5 min, RT). The cells were washed with PBS (3×) and imaged by confocal laser scanning microscopy (LSM 880, Carl Zeiss, Germany) using an oil immersion objective (Zeiss, 63× Plan-Apochromat, NA 1.4). Micrographs were recorded using a 633 nm HeNe laser to visualize Atto647-encapsulating polymersomes (detection range: 638–759 nm), a 561 nm DPSS 5561–10 laser for Alexa Fluor 555 (detection range: 519–644 nm), and a UV laser for Hoechst 33342 detection (detection range: 426–520 nm). The micrographs were analyzed using the ZEN 3.2 software (blue edition), the ImageJ software for uptake calculations and superimpose reconstructions, and Imaris software (Bitplane) for 3D reconstructions.

### Endosomal Escape Assay

For the endosomal escape of our CNCs, we performed calcein assays. HepG2 cells (30.000 cells/well) were plated in the wells of an ibidi 8-well chambered glass bottom coverslip (Vitaris, Switzerland) and cultured for 24 h. The next day, they were placed on ice for 10 min, followed by removal of the medium and addition of a fresh, cold medium containing calcein (250 μM) and polymersomes (0.3 mg mL^–1^). Cells were incubated on ice for further 30 min and returned to the incubator (5% CO_2_, 37 °C) for 4 h. Followed the incubation, cells were washed with PBS (3×) and phenol-free DMEM (200 μL) was added to each well. Cells were imaged under CLSM (λ_ex_ = 488 nm, argon laser, λ_em_ = 498–543 nm), and micrographs were processed using the ZEN Blue software (v.3.2, Carl Zeiss Microscopy GmbH) and ImageJ.

### Hymecromone Production

HepG2 and HeLa S3 H2B-GFP cells were seeded separately at a concentration of 5000 cells per well in a 96 well plate (100 μL). After 24 h, the medium was removed and replaced fresh DMEM mixed with nanocompartments in PBS (0.3 mg mL^–1^) and free enzymes ([GUS] 13 μg mL^–1^, [GOx] 97 μg mL^–1^) or PBS. The cells were cultured at 37 °C for another 24 h. The next day, cells were washed with PBS, and fresh DMEM was added containing 4-MUG (500 μM) or the equivalent amount of PBS. The fluorescence (λ_ex_: 365 nm, λ_em_: 445 nm) was monitored at several time points for 24 h on a SpectraMax id3 microplate reader (Molecular Devices, USA). For the estimation of hymecromone production, the GUS-GOx-CNCs-Gly incubated HepG2 cells were diluted (50×) at 24h and their fluorescence intensity was measured. Based on constructed calibration curves of 0, 2, 4, 6, 8, 10 μM 4-MU in DMEM, the produced hymecromone was calculated.

### Detection of H_2_O_2_

HepG2 and HeLa S3 H2B-GFP cells were seeded at a concentration of 30 000 and 20 000 cells (200 μL), respectively in each well of an ibidi 8-well chambered glass bottom coverslip (Vitaris, Switzerland). After 24 h, the medium was replaced with fresh DMEM containing either GUS-GOx-CNCs (0.3 mg mL^–1^), GUS-GOx-CNCs (0.3 mg mL^–1^), cGUS-GOx-CNCs (0.3 mg mL^–1^), empty polymersomes (0.3 mg mL^–1^), free enzymes ([GUS] 13 μg mL^–1^, [GOx] 97 μg mL^–1^) or the equivalent amount of PBS. Following a 24-h incubation, cells were washed, and the intracellularly produced H_2_O_2_ was detected using a ROS detection assay kit according to manufacturer’s instructions. As a positive control, untreated cells were incubated with *tert*-butyl hydroperoxide (inducer, provided by manufacturer) in ROS assay buffer for 1 h, 37 °C. Next, all samples were incubated with 2′,7′-dichlorodihydrofluorescein diacetate in ROS assay buffer (ROS label, provided by manufacturer, 150 μL/well) for 45 min, 37 °C, following a washing step with ROS assay buffer. Cells were imaged in ROS assay buffer (200 μL/well) under a CLSM (Argon laser, λ_ex_ = 488 nm, detection range 499–573 nm). Micrographs were analyzed using ZEN Blue software (v3.2, Carl Zeiss Microscopy GmbH) and ImageJ.

### Cell Viability Assay

HepG2 and HeLa S3 H2B-GFP cell viability was evaluated by CellTiter 96 AQueous One solution cell proliferation assay (MTS) following the supplier’s protocol. Briefly, cells were seeded at a concentration of 5000 cells per well in a 96 well plate (100 μL). After 24 h, the medium was removed and replaced fresh DMEM mixed with nanocompartments in PBS (0.3 mg mL^–1^), free glycooligomer (0.06 mg mL^–1^), free enzymes ([GUS] 13 μg mL^–1^, [GOx] 97 μg mL^–1^) or PBS. The cells were cultured at 37 °C for another 24 h. The MTS reagent (10 μL) was added to each well. Following a 2 h incubation at 37 °C, absorbance was measured at 490 nm using a SpectraMax plate reader. The data was normalized to PBS treated control cells after background absorbance removal.

For evaluating the cell viability after the addition of 4-MUG, cells were seeded and incubated with nanocompartments, free enzymes, and PBS as described above. After 24 h of incubation, cells were washed, and fresh DMEM was added containing 4-MUG (500 μM) or the equivalent amount of PBS and returned to incubator. The next day, the MTS cell proliferation assay was conducted as described above.

### Statistical Analysis

For comparative analysis, independent two-tailed *t* tests were used. *p* < 0.05 was considered statistically significant. The significance level was indicated by asterisks: *p* < 0.05 (*), *p* < 0.01 (**), *p* < 0.001 (***), *p* < 0.0001 (****).

## Results and Discussion

### Development of Glycooligomer-Functionalized Polymersomes

In order to generate polymersomes with functional groups exposed for the attachment of targeting moieties, we used a mixture of amphiphilic diblock copolymers, PDMS_25_-*b*-PMOXA_10_ and PDMS_22_-*b*-PMOXA_8_-OEG_3_-N_3_ (in a 1:1 molar ratio, Figures S1 and S2).^13,30,31^ The copolymers consisting of hydrophobic PDMS and hydrophilic PMOXA blocks are known for their biocompatibility, nontoxicity and stealth properties that are required for biomedical applications.^57−60^ The azide-functionalized diblock copolymer PDMS_22_-*b*-PMOXA_8_-OEG_3_-N_3_ served for covalent attachment of glycooligomer tethers. First, polymersomes (hereinafter referred to nanocompartments without encapsulated enzymes) were formed by film rehydration using phosphate buffered saline (PBS) solution and subsequently, extruded 21 times through a 100 nm polycarbonate membrane. The supramolecular assemblies were then decorated with a glycooligomer, specifically designed to interact selectively with mannose-binding lectins.^61^ The synthesis of the glycooligomer followed a previously reported procedure (elaborated in detail in Figures S3–S8).^48^ To decorate the polymersomes with glycooligomer tethers, we used the copper-catalyzed azide–alkyne cycloaddition (CuAAC) reaction between the alkyne end of glycooligomers and the azide moieties present on the outer membrane of polymersomes (sodium l-ascorbate 10 mol %, CuSO_4_·5H_2_O 1 mol %). The glycosylated polymersomes were purified by size exclusion chromatography (SEC). Considering the potential biomedical applications of our catalytic nanocompartments, we performed colorimetric copper detection assays to evaluate the level of residual copper. The amount of free copper remaining after purification was well below physiological thresholds and, therefore, not expected to be toxic (Figure S9).^62^

**Figure 2:**
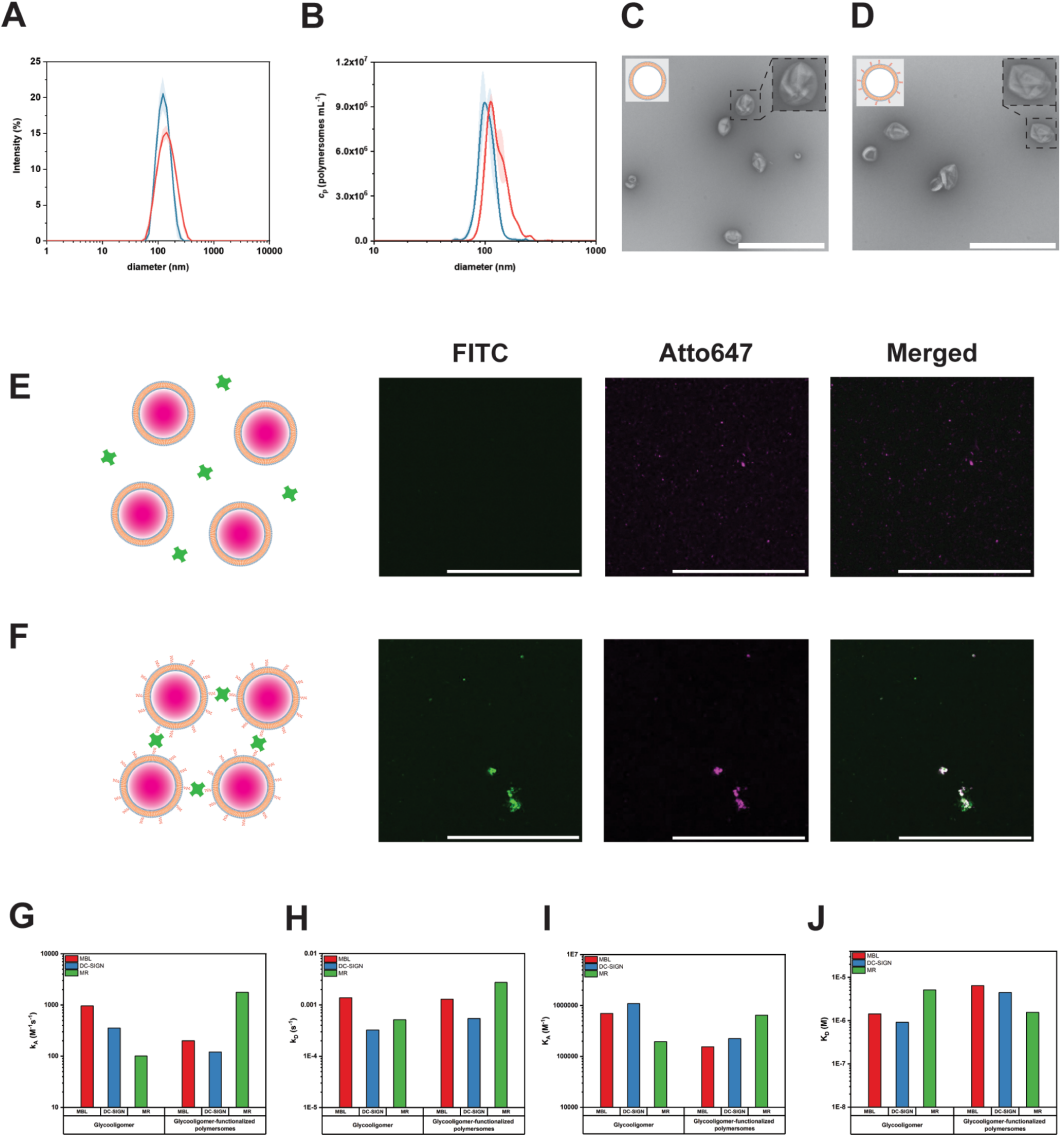
Development of glycooligomer-decorated polymersomes. (A) DLS intensity size distribution of nonfunctionalized (blue) and glycooligomer-functionalized polymersomes (red); (curves represent mean ± s.d. of three independent measurements) (B) NTA concentration size distribution of nonfunctionalized (blue) and glycooligomer-functionalized polymersomes (red); (curves represent mean ± s.d. of three independent measurements). TEM micrographs of (C) nonfunctionalized and (D) glycooligomer-functionalized polymersomes (Scale bar: 1000 nm, 2× zoom-in of selected polymersomes). CLSM micrographs of (E) nonfunctionalized and (F) glycooligomer-functionalized Atto647-loaded polymersomes upon incubation with FITC-ConA. Scale bar: 20 μm. Kinetic parameters obtained from SPR binding curves by 1:1 Langmuir fitting for glycooligomer and glycooligomer-functionalized polymersomes with lectins MBL, DC-SIGN, and MR. (G) Association rate constants (*k*_A_), (H) dissociation rate constants (*k*_D_), (I) association constants (*K*_A_), and (J) dissociation constants (*K*_D_).

Their morphological characterization was performed using a combination of dynamic and static light scattering (DLS/SLS), nanoparticle tracking analysis (NTA) and transmission electron microscopy (TEM, Figure 2A–D). An average diameter of 122 ± 40 nm for the nonfunctionalized polymersomes and 135 ± 54 nm for the glycosylated ones was obtained by DLS (Figure 2A). The slight increase in the apparent diameter of functionalized polymersomes was also observed by NTA, and it can be attributed to the presence of glycooligomer tethers on their surface (Figure 2B and Table S1), as previously reported for other targeting molecules attached to polymersomes.^25^ The radius of gyration (*R*_g_) values of 66 ± 4 and 79 ± 13 nm, respectively, were obtained by SLS (Table S1 and Figure S10). The ratio of *R*_g_ to the hydrodynamic radius (*R*_h_, obtained by the DLS profile, Figure S8) (ρ factor) of around 1 for both non- and glycooligomer-functionalized polymersomes indicated the typical vesicular structure and preservation of polymersome morphology after attachment of the targeting molecules (Table S1). The size distribution, polymersome morphology, and lack of aggregation for both non- and glycooligomer-functionalized polymersomes were further corroborated by TEM micrographs (Figures 2C,D and S11A,B). The integrity of non- and glycooligomer-functionalized polymersomes was additionally evaluated after the encapsulation of the fluorescent Atto647 dye in their cavities during the self-assembly process. Diameters of 137 ± 39 nm for the glycooligomer-functionalized and 128 ± 40 nm for nonfunctionalized Atto647-loaded polymersomes indicated that the encapsulation of the dye neither affected the size nor the morphology of the polymersomes (Table S1).

The pore-forming peptide, melittin, was added to the rehydration buffer in order to be inserted and permeabilize the membrane of the resulting polymersomes and to allow the essential molecular through-flow. To assess its insertion into the polymersomes’ membrane, a series of fluorescence correlation spectroscopy (FCS) measurements were performed on non- and glycooligomer functionalized polymersomes permeabilized with various concentrations of Cy5-labeled melittin (25, 50, or 75 μM). The diffusion times corresponding to polymersomes confirmed the association of melittin with the polymersomes’ membrane (Table S2). By correlating the molecular brightness values of free Cy5-melittin and Cy5-melittin polymersomes, we estimated the average number of pores per polymersome, which ranged from 133 to 242 (Table S2). The concentration of melittin was chosen at 50 μM for further studies, as the number of inserted pores did not significantly change between 50 and 75 μM of initial melittin concentration (Table S2). The comparable number of melittin pores between nonfunctionalized (242 ± 28 pores/polymersome) and glycoligomer-functionalized (220 ± 16 pores/polymersome) polymersomes indicates that surface functionalization with glycooligomers neither affected the accessibility of the melittin pores nor induced aggregation.

The covalent attachment of glycooligomer tethers was followed by clustering experiments using concanavalin A labeled with fluorescein isothiocyanate (FITC-ConA) for detection under a fluorescence microscope.^63,64^ Confocal laser scanning microscopy (CLSM) analysis revealed that only glycooligomer-functionalized polymersomes, loaded with the Atto647 fluorescent dye, formed clusters with FITC-labeled ConA (Figure 2E,F). This was evidenced by a calculated Pearson’s colocalization coefficient of 0.778 ± 0.071 for the two dyes present in the system (Figure 2F). In contrast, nonfunctionalized polymersomes exhibited no cluster formation, as indicated by the low Pearson’s coefficient of 0.012 ± 0.009, emphasizing the importance of functionalization in the process of clustering (Figure 2E). As determined by glycooligomer absorbance at 250 nm (absorbance peak of glycooligomer), approximately 30% (equivalent to 0.23 mM) of the initial amount of glycooligomer was successfully conjugated onto the polymersomes (Figure S12). By correlating the amount of glycooligomer with the maximum polymersome concentration, we estimated an average number of 125 ± 5 glycooligomers per polymersome.

To further explore the functional glycosylation of polymersomes and evaluate their binding properties with carbohydrate-binding proteins (lectins), we used surface plasmon resonance (SPR) (Figures 2G–J and S13). Human lectins such as mannose-binding lectin (MBL), mannose receptor 1 (MR, CD206), dectin-1 (CD369), and dendritic cell-specific intercellular adhesion molecule-3-grabbing nonintegrin (DC-SIGN, CD209), known for their role in the innate immune response and various cancers were selected as binding ligands to study the interaction with our glycooligomer.^38,65−68^ The acquired binding curves are a clear indication of the glycooligomer ability to specifically interact with MBL, as evident from the very sharp increase in the signal intensity upon sample injection (Figure S13). This was followed by a plateau, suggesting saturation of the chip-bound lectins, and a subsequent signal decrease upon buffer injection due to carbohydrates disassociating from the lectins. Glycooligomer-functionalized polymersomes also showed a strong binding to MBL. However, the curve shapes during the association phase were less distinct due to the higher molecular weight of the glycooligomer-functionalized polymersomes (Figure S13). These findings underpinned by the calculated association constants (*K*_A_, Figure 2I), revealed an approximately 4.5-fold stronger binding of the free glycooligomer compared to glycooligomer-decorated polymersomes. Interestingly, this trend was also observed for DC-SIGN (Figure 2G–J), whereas, in the case of MR, the functionalized polymersomes showed faster binding kinetics compared to the free ligand, with a difference in *K*_A_ of about 3.2-fold. Steric hindrance posed by the larger polymersomes appeared to predominantly affect the slower binding kinetics observed for MBL and DC-SIGN. Both the free glycooligomer and the glycooligomer-functionalized polymersomes bound weakly to dectin-1, a lectin known to recognize β-glucans, indicating a certain level of off-target interaction with mannose moieties (Figure S13).^69^ Nonfunctionalized polymersomes showed weak, nonspecific binding to MBL, dectin-1, MR, and DC-SIGN with significantly lower response levels (Figure S13).

### Development of Glycooligomer-Functionalized Dual-Enzyme Loaded Catalytic Nanocompartments

**Figure 3:**
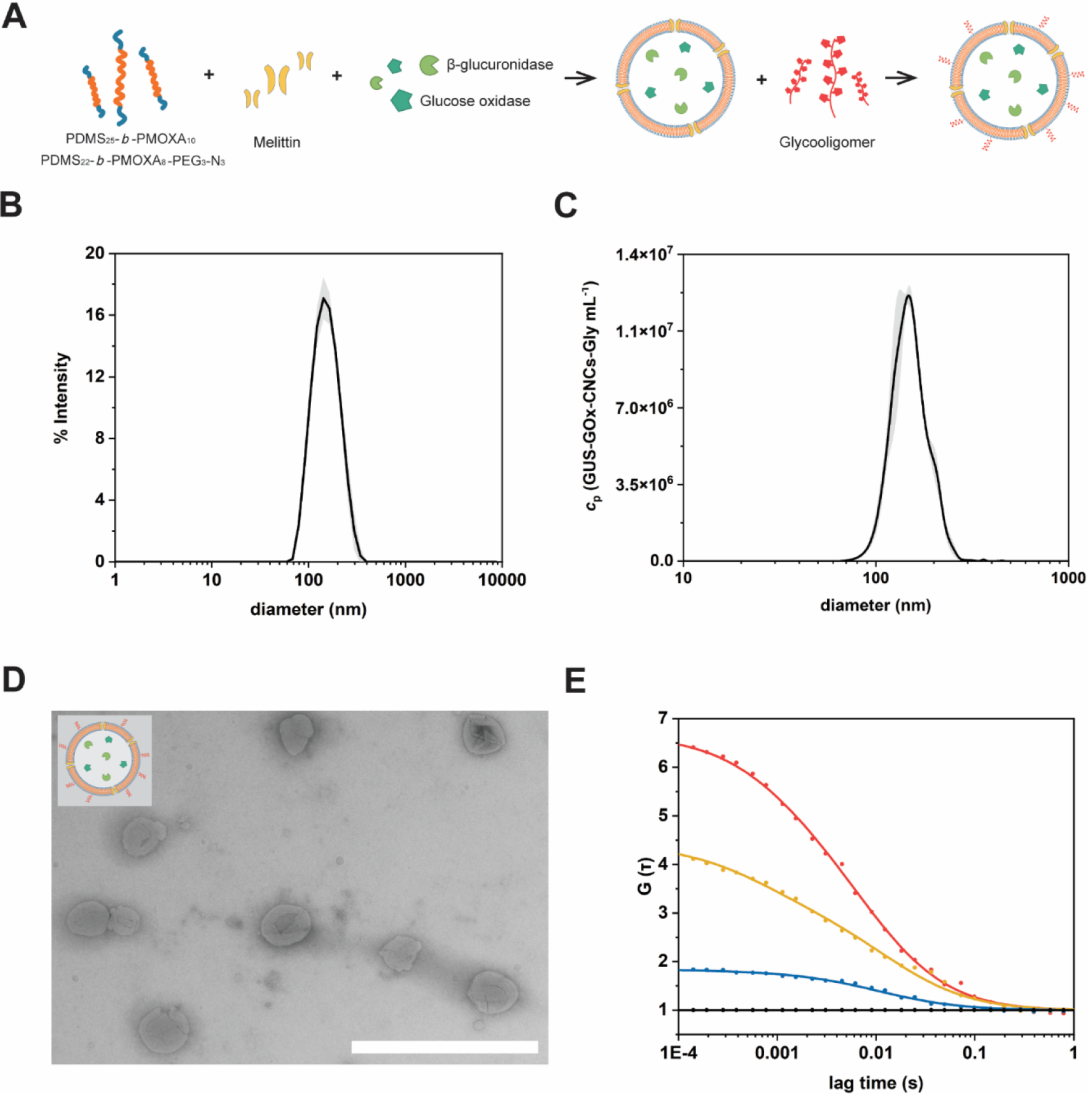
Development of GUS-GOx-CNCs-Gly. A. Schematic representation illustrating the formation of GUS-GOx-CNCs-Gly. B. DLS intensity size distribution (curves represent mean ± s.d. of three measurements) C. NTA concentration and size distribution (curves represent mean ± s.d. of three measurements) D. TEM micrograph of GUS-GOx-CNCs-Gly (Scale bar: 1000 nm) E. FCS curves of GUS-GOx-CNCs-Gly (yellow: channel for Atto488-GUS, red: channel for Atto633-GOx) and FCCS curves of GUS-GOx-CNCs-Gly (blue) and free Atto488-GUS/Atto633-GOx (black). Symbols: raw data, Lines: fitted curves.

Our catalytic nanocompartments were also produced by film rehydration in the presence of a mixture of β-glucuronidase (GUS, 0.5 mg mL^–1^), glucose oxidase (GOx, 0.5 mg mL^–1^), and melittin (50 μM) in PBS solution (Figure 3A). These conditions of encapsulation were chosen based on the similar catalytic activity of the enzymes. The resulting CNCs (GUS-GOx-CNCs) were incubated with proteinase K for deactivation of nonencapsulated enzymes, purified, and functionalized with the glycooligomer (GUS-GOx-CNCs-Gly), in similar condition to polymersomes. The size analysis of GUS-GOx-CNCs-Gly revealed diameters of 152 ± 54 nm (by DLS; Figure 3B) and 137 ± 38 nm (by NTA; Figure 3C). A slight increase in the net charge of glycooligomer-functionalized CNCs (5.3 ± 1.7 mV) compared to nonfunctionalized CNCs (3.0 ± 0.6 mV) was observed by measuring their z-potential. The coencapsulation of enzymes and simultaneous insertion of melittin did not affect the self-assembly process and their size distribution. With a calculated ρ factor of around 1, the morphology of CNCs remained vesicular as of the empty polymersomes (Figure S14). TEM micrographs indicated a vesicular, collapsed architecture of the CNCs, typical of PDMS-*b*-PMOXA polymersomes (Figure 3D).^13,31^ The size distribution of GUS-GOx-CNCs-Gly was also confirmed by measuring their diameter in TEM micrographs (Figure S11C). These results taken together indicate that the encapsulation of two enzymes and insertion of melittin had no effect on the self-assembly, size distribution, and morphology of our CNCs.

The encapsulation of both enzymes in CNCs was evaluated by fluorescence correlation and cross-correlation spectroscopy (FCS/FCCS, Figure 3E). GUS was labeled with Atto488 (τ_D_ free Atto488 29 ± 4 μs, τ_D_ GUS-Atto488 250 ± 92 μs, 1.7 ± 0.1 dyes/enzyme, Figure S15), and GOx was labeled with Atto633 (τ_D_ free Atto633 57 ± 6 μs, τ_D_ GOx-Atto488 424 ± 32 μs, 1.5 ± 0.1 dyes/enzyme, Figure S15). The FCS autocorrelation curves indicated the successful encapsulation of GUS (4 ± 2 molecules) and GOx (7 ± 4 molecules) within the CNCs based on the significant change in diffusion time (τ_D_ GUS-GOx-CNCs-Gly 6900 ± 3940 μs). FCCS can be utilized to study the association between two different fluorophores when their signals correlate.^70^ Therefore, we used FCCS analysis to investigate the coencapsulation of the two enzymes in the cavities of CNCs, as GUS and GOx were fluorescently labeled with different fluorophores. The increased cross-correlation (Figure 3E; blue curve) of GUS and GOx when they were encapsulated in CNCs in comparison to the free enzymes (Figure 3E; black curve) indicated their coencapsulation in the CNCs’ cavities. Correlating the number of both encapsulated enzymes to the concentration of CNCs measured by NTA, we calculated an enzyme encapsulation efficiency of 16% ± 8% for GUS and 11% ± 6% for GOx. We also used the bicinchoninic acid (BCA) assay for quantification of total amount of protein in our CNCs (Figure S16). A total 24% ± 7% of the initial amount of enzyme (both GUS and GOx) was obtained, which is in line with the calculated sum of the encapsulation efficiency. Applying the Stokes–Einstein equation together with the FCS diffusion times, a calculated CNC size of 144 ± 64 nm in diameter was obtained, which is in agreement with the values obtained from DLS, SLS, NTA, and TEM measurements (Figures 3, S14 and S11C).

### Parallel Production of Hymecromone and H_2_O_2_ by CNCs

**Figure 4:**
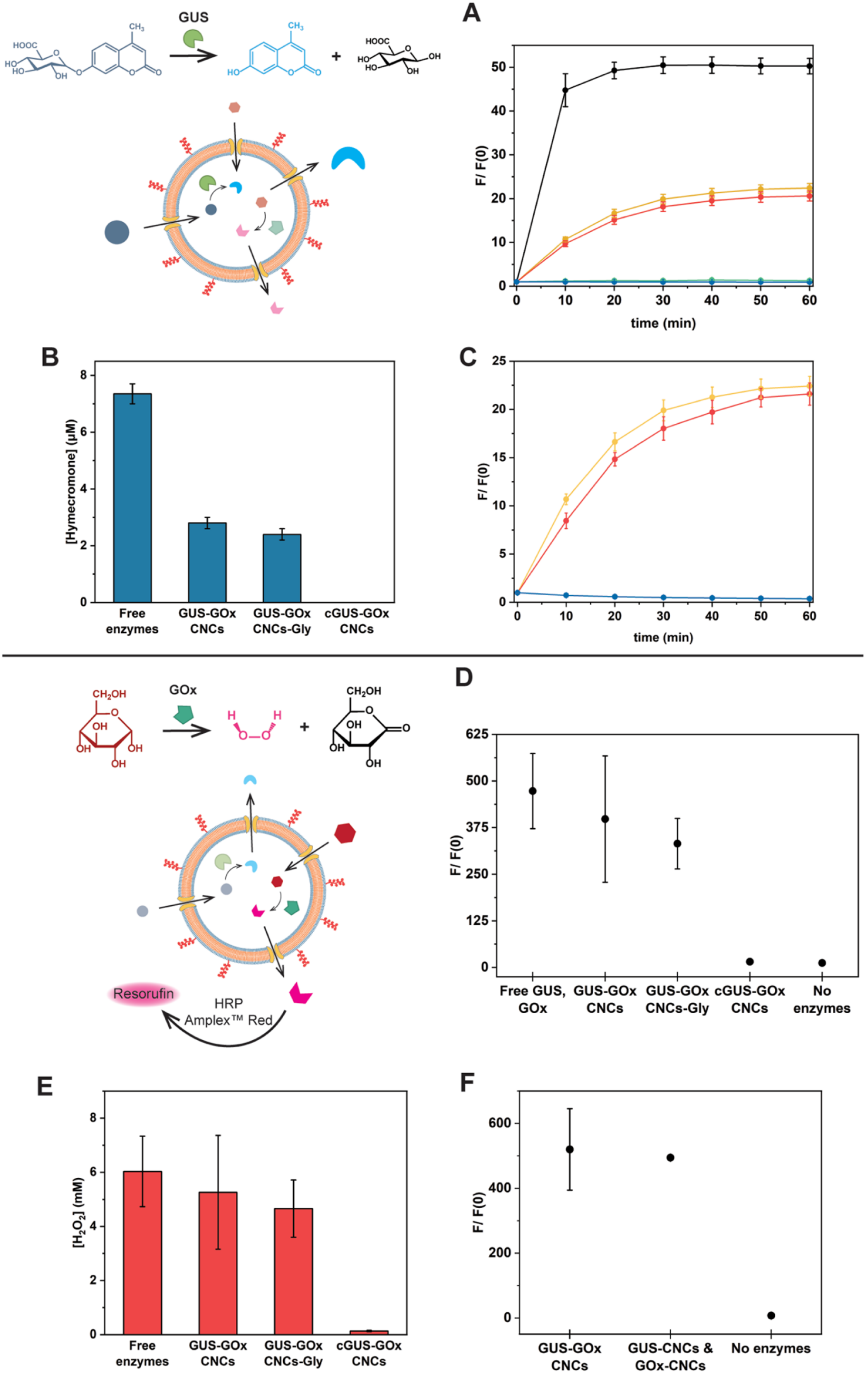
Parallel enzymatic reactions in PBS containing 50% DMEM Phenol Red free with 10% FBS at 37 °C A. 4-MUG (10 μM) conversion to 4-MU, black: free enzymes, yellow: GUS-GOx-CNCs, red: GUS-GOx-CNCs-Gly, green: nonpermeabilized CNCs, blue: reaction mixture without enzymes. Mean ± s.d. of 3 experiments. B. Amount of hymecromone produced after 60 min. Mean ± s.d. of 3 experiments. C. 4-MUG (10 μM) conversion to 4-MU, yellow: GUS-GOx-CNCs, red: GUS-CNCs, blue: reaction mix without enzymes. Mean ± s.d. of 3 repetitions. D. Conversion of Amplex Red (10 μM) into resorufin after 2 h. Mean ± s.d. of 3 experiments. E. Amount of H_2_O_2_ produced after 2 h. Mean ± s.d. of 3 experiments. F. Amplex Red (10 μM) conversion to resorufin after 2 h by GUS-GOx-CNCs and GOx-CNCs. Mean ± s.d. of 3 repetitions. Error bars might be smaller than the symbol.

In contrast to previously reported enzyme-bearing CNCs for cascade reactions,^71−75^ our CNCs were specifically designed to facilitate parallel production of hymecromone and H_2_O_2_. When provided with its glucuronide conjugate, GUS produces the drug hymecromone, while GOx consumes the existing glucose in the medium and produces H_2_O_2_ (Figure 4). Free and encapsulated enzymes were assessed for their enzymatic activity in a PBS solution comprising 50% Dulbecco’s modified Eagle medium (DMEM) Phenol Red free with 10% fetal bovine serum (FBS). This formulation was selected to mimic the cell culture environment, as it is essential for evaluating the therapeutic potential of our CNCs. First, we monitored the activity of GUS by tracking the conversion of hymecromone glucuronide (4-MUG) into hymecromone (λ_em_ 445 nm) over 60 min (Figure 4A). Upon the addition of 4-MUG (10 μM) to free GUS, the fluorescence levels corresponding to hymecromone rapidly reached a plateau within 20 min (Figure 4A, black). GUS-GOx-CNCs and GUS-GOx-CNCs-Gly gradually reached a plateau at around 45 min (Figure 4A, yellow and red). Comparing the slopes of the linear part of the reaction, which describe the rate of 4-MU production, we observed a decrease in their value for encapsulated GUS (0.78 ± 0.11 for GUS-GOx-CNCs and 0.71 ± 0.09 for GUS-GOx-CNCs-Gly), compared to free enzyme (4.37 ± 0.10). This behavior is characteristic of pore-permeabilized enzyme-encapsulating nanocompartments, where the diffusion of substrates and products to and from the cavities might affect the velocity of the reaction.^9,13^ As expected, nonpermeabilized CNCs (cGUS-GOx-CNCs) demonstrated a minimal hymecromone fluorescence level over time because the polymersome membrane did not allow the diffusion of substrate into the cavity (Figure 4A, green). No increase in fluorescence was observed for 4-MUG alone (Figure 4A, blue), revealing that in the absence of GUS, hydrolysis of the ether bond and production of 4-MU do not occur. According to the 4-MU calibration curve, free GUS generated 7.4 ± 0.4 μM, while GUS-GOx-CNCs produced 2.8 ± 0.2 μM and GUS-GOx-CNCs-Gly 2.4 ± 0.2 μM of hymecromone (Figures S17A and 4B). These differences in hymecromone production between free and encapsulated GUS are attributed to the molecular crowding caused by FBS and the diffusion of substrates and products through the CNCs’ membrane.^13^ In PBS containing 10 μM 4-MUG and 12.5 mM glucose, the fluorescence signal rapidly increased for free GUS, reaching a plateau in 10 min (Figure S18A). On the contrary, the fluorescence signal continued to increase over 35 min for GUS-GOx-CNCs and GUS-GOx-CNCs-Gly, revealing that FBS affects the kinetics of the free enzyme, but not those of the confined one (Figure S18A).^13^ To investigate whether the coencapsulation of GUS with GOx had an influence on the GUS reaction kinetics, we confined the enzymes into the cavities of separate nanocompartments produced in similar conditions (GUS-CNCs and GOx-CNCs, Figure 4C). As evident by the similarity between the increase in the fluorescence of GUS-GOx-CNCs and GUS-CNCs, as well as the slopes of the reactions (0.69 ± 0.03 for GUS-CNCs), the enzymatic activity of GUS was not affected by its coencapsulation with GOx, thus these reactions are bioorthogonal. This represents a crucial requirement when dual-enzyme CNCs are developed to not compromise the in situ reactions.

In parallel, the available glucose (12.5 mM) was consumed by the second enzyme, GOx, resulting in the production of H_2_O_2_. To accurately monitor and quantify the generated H_2_O_2_, we employed the Amplex Red (AR) assay, which in the presence of horseradish peroxidase (HRP) stoichiometrically reacts with H_2_O_2_ (Figure 4D). HRP and AR were added to the solution of CNCs and AR reacted with the H_2_O_2_ released from the CNCs producing fluorescent resorufin (λ_em_ 590 nm). For free GOx in solution, the resorufin fluorescence signal increased approximately 470 times, equivalent to 6.0 ± 1.3 mM of H_2_O_2_ according to the resorufin reference curve (Figures 4D,E and S17B). For GUS-GOx-CNCs and GUS-GOx-CNCs-Gly, the fluorescence increased by 400 and 330 times, respectively, corresponding to 5.2 ± 2.1 mM H_2_O_2_ for GUS-GOx-CNCs and 4.7 ± 1.1 mM H_2_O_2_ for GUS-GOx-CNCs-Gly. Comparable amounts of H_2_O_2_ were produced by free GOx, GUS-GOx-CNCs, and GUS-GOx-CNCs-Gly due to the high excess of glucose within the system. On the contrary, a minimum fluorescence signal was detected for nonpermeabilized CNCs and AR alone, associated with the autoxidation of AR. The impact of FBS on the kinetics of GOx was also evaluated. When the reactions were conducted in PBS containing 10 μM 4-MUG and 12.5 mM glucose, free GOx showed a 625-fold increase in resorufin fluorescence, whereas GUS-GOx-CNCs and GUS-GOx-CNCs-Gly showed increases of 520 and 458 times, respectively. This difference indicates the crowding effect of the protein-rich FBS (Figure S18B). Co-encapsulation with GUS did not affect the reaction kinetics of GOx, as indicated by the similar changes in resorufin fluorescence between coencapsulated and separately encapsulated enzymes (Figure 4F). Importantly, the enzyme reaction kinetics (Figure 4A,D) and the catalytic efficiencies (Figure 4B,E) of both GUS and GOx were similar in GUS-GOx-CNCs-Gly and GUS-GOx-CNCs. Therefore, the presence of glycooligomers on the outer membrane did not restrict the diffusion of substrates and products to and from the CNCs’ cavities. Our CNCs were stored for up to 2 months at 4 °C in PBS. Remarkably, they preserved their size as measured by DLS and retained their GUS and GOx activity, which is an important aspect in nanosystems developed for therapeutic applications (Figure S19).

### Cell Targeting, Uptake, and Endosomal Escape of Glycooligomer-Functionalized Nanocompartments

**Figure 5:**
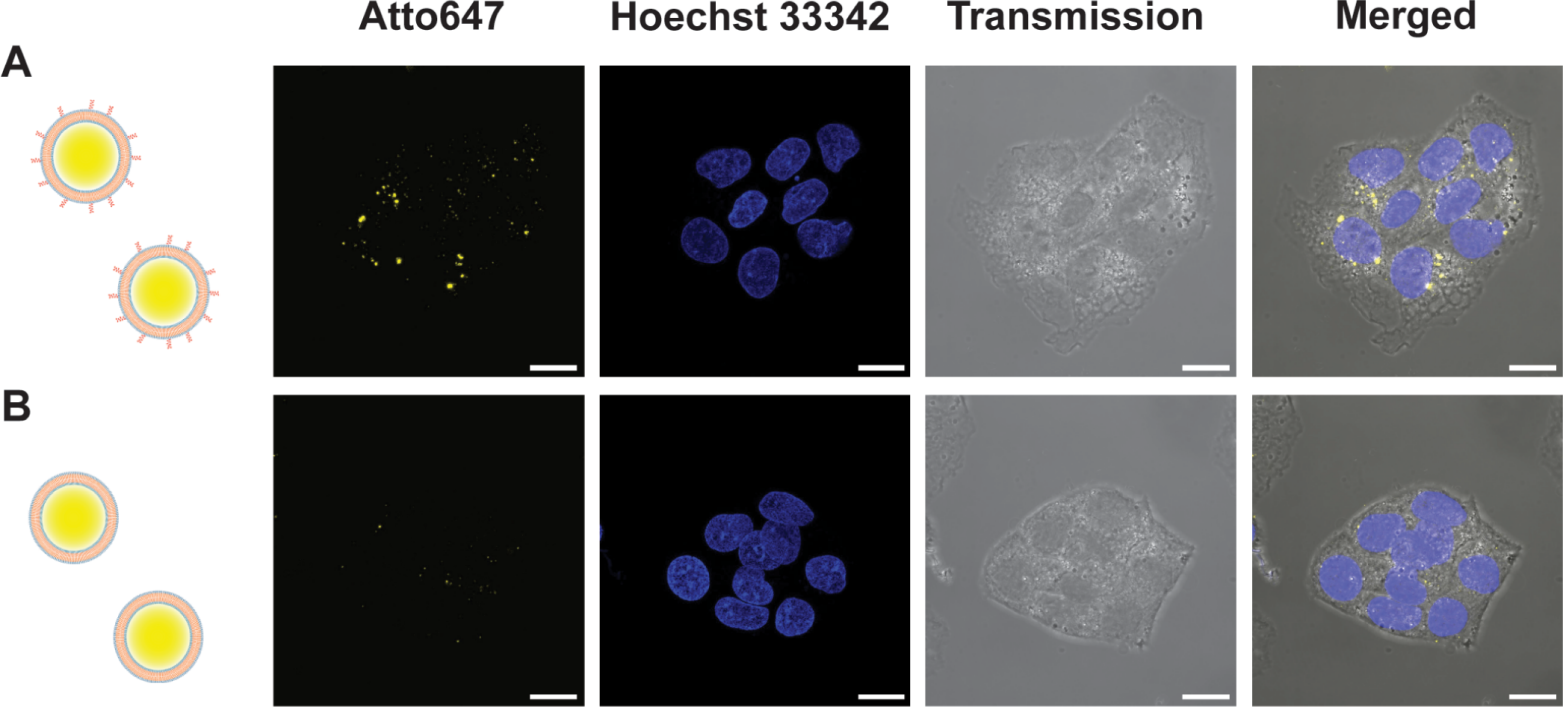
Uptake of Atto647-encapsulating polymersomes by HepG2 cells. Superimposed CLSM micrographs of multiple confocal sections. A. HepG2 cells incubated with glycooligomer-functionalized polymersomes. B. HepG2 cells incubated with nonfunctionalized polymersomes. Yellow: polymersomes, Atto647; blue: nuclei, Hoechst 33342. Scale bar: 10 μm.

**Figure 6:**
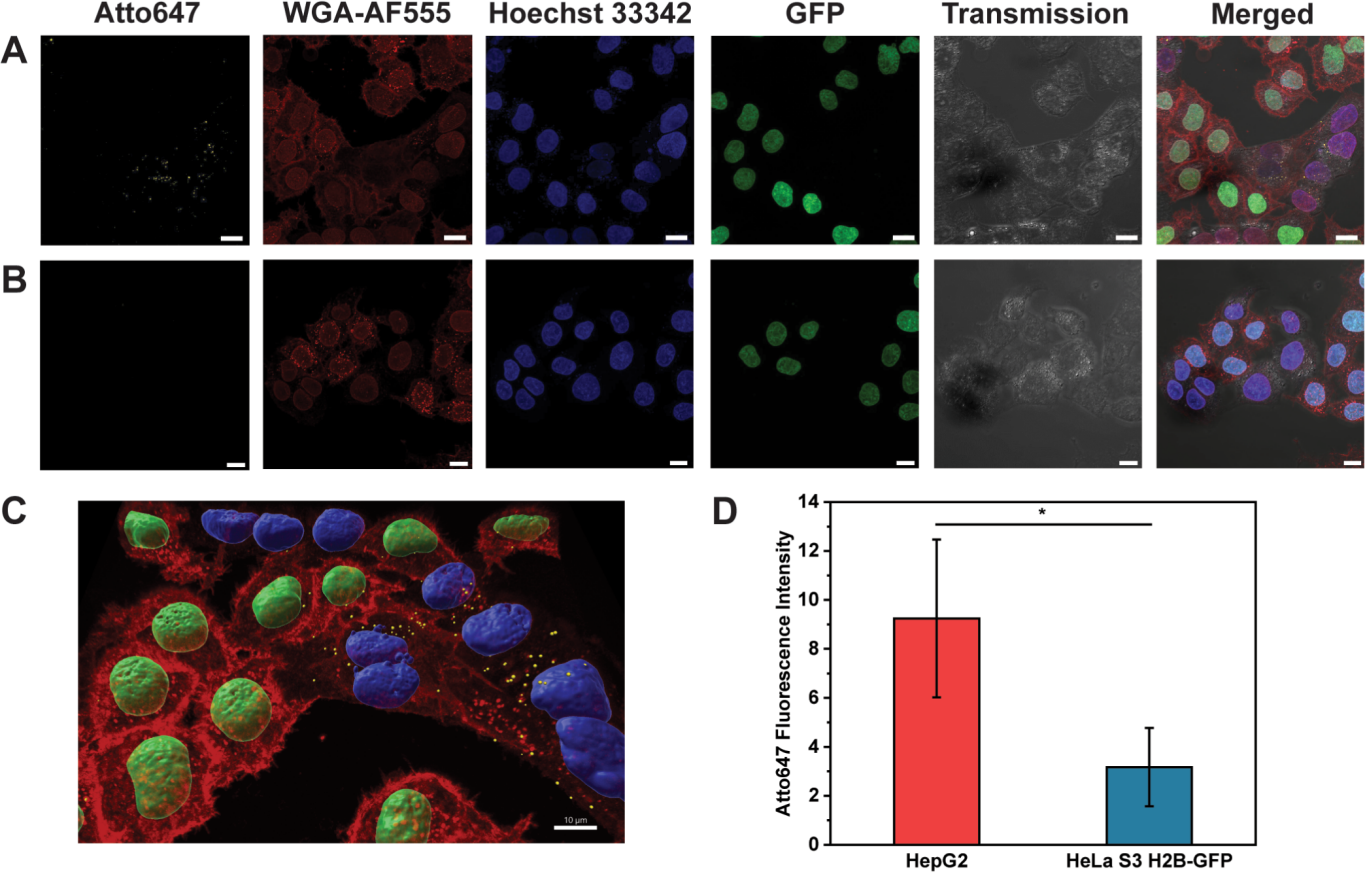
Uptake of Atto647-encapsulating polymersomes by HepG2 and HeLa S3 H2B-GFP cells. Superimposed CLSM micrographs of multiple confocal sections. A. Cells incubated with glycooligomer-functionalized polymersomes. B. Cells incubated with nonfunctionalized polymersomes. C. 3D reconstructions of multiple confocal sections of HepG2 and HeLa cocultured cells incubated with glycooligomer-functionalized polymersomes. D. Total fluorescence intensity of Atto647 analyzed by CLSM micrographs. Yellow: polymersomes, Atto647; red: cell membranes, Atto555-WGA; blue: nuclei, Hoechst 33342; green: HeLa S3 H2B-GFP nuclei, GFP. Scale bar: 10 μm.

Liver cells are known to express a high level of mannose receptors, facilitating receptor-assisted endocytosis and internalization of the mannose-containing molecules.^76^ To assess the targeting efficiency and uptake potential of glycooligomer-functionalized polymersomes, we used two distinct cell lines: HepG2 (a liver-derived cancer cell line overexpressing mannose-binding lectins) and HeLa cells (a cervix-derived cancer cell line with low expression of mannose-binding lectins) (Figures 5, 6 and S20).^34,35,51−53^ First, we studied the ability of conjugated glycooligomer to promote polymersome uptake in HepG2 cells (Figures 5 and S20A). Cells were incubated for 24 h with either glycooligomer-functionalized or nonfunctionalized Atto647-containing polymersomes and were imaged using CLSM. Analysis of CLSM micrographs in HepG2 cells showed a considerable increase in the uptake of glycooligomer-functionalized polymersomes compared to nonfunctionalized ones, located in the cells’ cytoplasm (Figure 5A,B). Quantification of the fluorescence intensity revealed a 4-fold increase (*p*-value < 0.01) for glycosylated polymersomes. To evaluate whether our polymersomes successfully escape the endosomes after cell uptake, we performed endosomal escape assays by coincubating HepG2 cells with a mixture of non- or glycooligomer-functionalized polymersomes and free calcein for 4 h. CLSM imaging indicated that glycooligomer-functionalized polymersomes had escaped the endosomes and were localized within the cytoplasm of cells, as indicated by the dispersed green cytosolic fluorescence (Figure S21). The higher endosomal escape observed with glycooligomer-functionalized polymersomes compared to nonfunctionalized ones correlates with their higher uptake level (Figure 5). These results are in agreement with previous studies demonstrating that the endosomal escape of nanoparticles in HepG2 cells occurs over a period of 3–14 h.^77−80^

To further explore the specificity in cell uptake of glycooligomer-decorated polymersomes, HepG2 and HeLa S3 H2B-GFP cells were cocultured and subsequently incubated with Atto647-loaded polymersomes for 24 h (Figures and6S20B). Analysis of CLSM micrographs revealed that glycooligomer-functionalized polymersomes were predominantly found in HepG2 cells (Figure 6A–C). Upon quantifying the fluorescence intensity corresponding to Atto647, we obtained that glycosylated polymersomes were 3 times (*p*-value < 0.05) more abundant in HepG2 cells in comparison to HeLa cells (Figure 6D). These findings underline the key role of glycooligomer functionalization in enabling specific cell uptake, particularly demonstrating the enhanced uptake of our catalytic nanocompartments in liver cells expressing high levels of mannose-binding lectins.

### Catalytic Nanocompartments in Cells – Parallel Reactions and a Synergistic Effect of Hymecromone and H_2_O_2_

**Figure 7:**
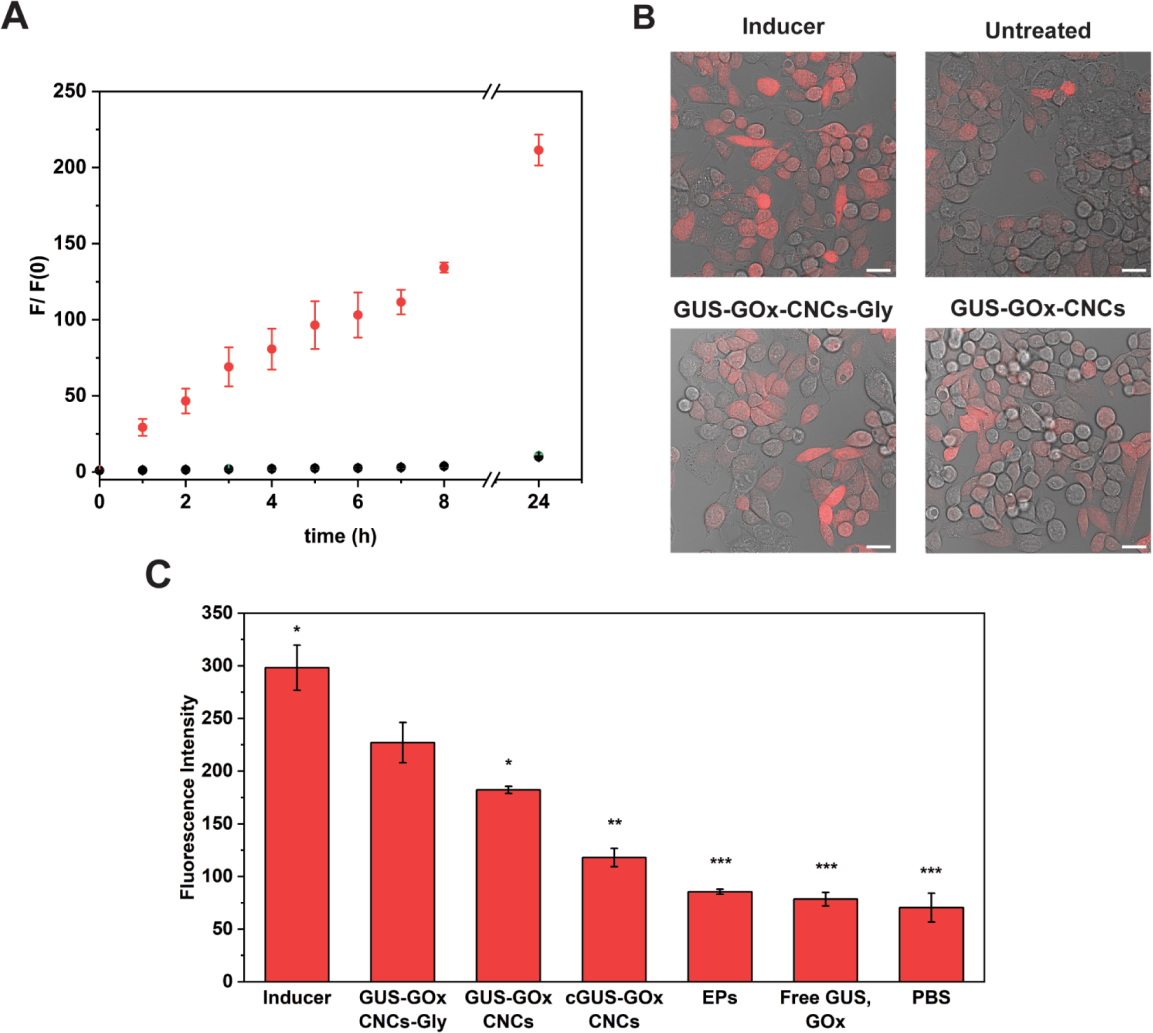
Parallel reactions by dual enzyme-loaded catalytic nanocompartments in HepG2 cells. A. Intracellular production of hymecromone after incubation with GUS-GOx-CNCs (yellow), GUS-GOx-CNCs-Gly (red), cGUS-GOx-CNCs (green), free GUS/GOx mixture (blue), or PBS (black) for 24 h. Cells were washed and exposed to a single dose of 500 μM 4-MUG. Fluorescence was recorded for 24 h. Graph shows mean ± s.d. of three experiments. B. CLSM micrographs of 2′,7′-dichlorodihydrofluorescein diacetate incubated cells. Red: ROS species, DCF. Scale bar: 20 μm. C. Total fluorescence intensity of DCF as analyzed by CLSM micrographs. Asterisks: statistical significance between GUS-GOx-CNCs-Gly and the corresponding column. Graph shows mean ± s.d. of three micrographs.

We evaluated the multifunctionality of the glycooligomer-functionalized catalytic nanocompartments in cells as an essential step to assess their potential (Figure 7). We first examined hymecromone production in HepG2 and HeLa S3 H2B-GFP cells after incubation with GUS-GOx-CNCs-Gly, GUS-GOx-CNCs, non- permeabilized CNCs, free GUS/GOx, or an equivalent volume of PBS for 24 h. After cell washing for removal of nonuptaken CNCs or enzymes, a fresh medium containing 4-MUG (500 μM) was added, and the increase in the hymecromone fluorescence signal was monitored for 24 h (Figures 7A and S22). At this concentration, hymecromone has shown an inhibitory effect on hyaluronan synthesis and induced cell death.^81,82^ In HepG2 cells, exposure to GUS-GOx-CNCs-Gly resulted in a progressive increase of the fluorescence signal, corresponding to 190 ± 23 μM of hymecromone at 24 h (Figure S23). In contrast, no fluorescence increase was observed in cells incubated with PBS, free GUS, GUS-GOx-CNCs, and nonpermeabilized CNCs. Proteins are large, hydrophilic molecules that cannot pass directly through the cell membrane and are degraded by extracellular proteases.^83−85^ Therefore, only when encapsulated inside catalytic nanocompartments, GUS is effectively shielded, thus prolonging the activity and facilitating the cellular uptake (Figure 7A). Notably, GUS-GOx-CNCs and GUS-GOx-CNCs-Gly in solution demonstrated a similar enzymatic activity (Figure 4), indicating the intracellular production of hymecromone specifically by the cells that have taken up the catalytic nanocompartments. However, in HeLa S3 H2B-GFP cells characterized by a lower expression of mannose-binding lectins, GUS-GOx-CNCs-Gly produced only 8 ± 2 μM of hymecromone, indicating a significantly lower uptake of the CNCs (Figures S22 and S23). When free GUS, GUS-GOx-CNCs, and cGUS-GOx-CNCs was added to HeLa S3 H2B-GFP cells, no increase in fluorescence corresponding to hymecromone production was observed. Similarly as in solution, drug production does not occur in the case of nonpermeabilized CNCs (Figure 7A).^13^

We then detected the intracellularly produced H_2_O_2_ in HepG2 and HeLa3S H2B-GFP cells using a cell-permeable 2′,7′-dichlorodihydrofluorescein diacetate (H2DCFDA) probe. This nonfluorescent probe, upon cleavage by intracellular esterases and ROS oxidation, is converted to fluorescent 2′,7′-dichlorofluorescein (DCF) (Figures 7B,C, S24 and S25). Total fluorescence analysis of HepG2 cells incubated with GUS-GOx-CNCs-Gly exhibited significantly higher DCF fluorescence intensity compared to cells incubated with GUS-GOx-CNCs (1-fold, *p*-value < 0.05), thus indicating higher intracellular ROS levels (Figures 7B,C and S24). These increased levels correlate with the enhanced cellular uptake and endosomal escape of glycooligomer-functionalized CNCs in HepG2 cells. Cells incubated with GUS-GOx-CNCs-Gly also showed significantly higher DCF fluorescence intensity compared to nonpermeabilized CNCs (cGUS-GOx-CNCs, 2-fold, *p*-value < 0.01) and free GUS/GOx (3-fold, *p*-value < 0.001), respectively, highlighting the importance of our permeabilized, glycooligomer-functionalized CNCs for intracellular uptake and activity. Moreover, GUS-GOx-CNCs-Gly induced significantly higher intracellular DCF fluorescence intensity compared to empty polymersomes (EPs, 3-fold, *p*-value < 0.001) and PBS (3-fold, *p*-value < 0.001), respectively. The DCF fluorescence intensity detected in untreated HepG2 cells is associated with their intrinsic ROS levels.^86^ These findings further underline the efficacy of our GUS-GOx-CNCs-Gly in inducing high intracellular ROS levels. In contrast, total DCF fluorescence analysis in HeLa S3 H2B-GFP cells did not reveal significant differences in intracellular ROS levels when incubated with CNCs or free enzymes (Figure S25). Similar to hymecromone production, free GOx and CNCs are not efficiently uptaken by HeLa S3 H2B-GFP cells, with nonpermeabilized CNCs bearing the extra constraint of an impermeable membrane.

**Figure 8:**
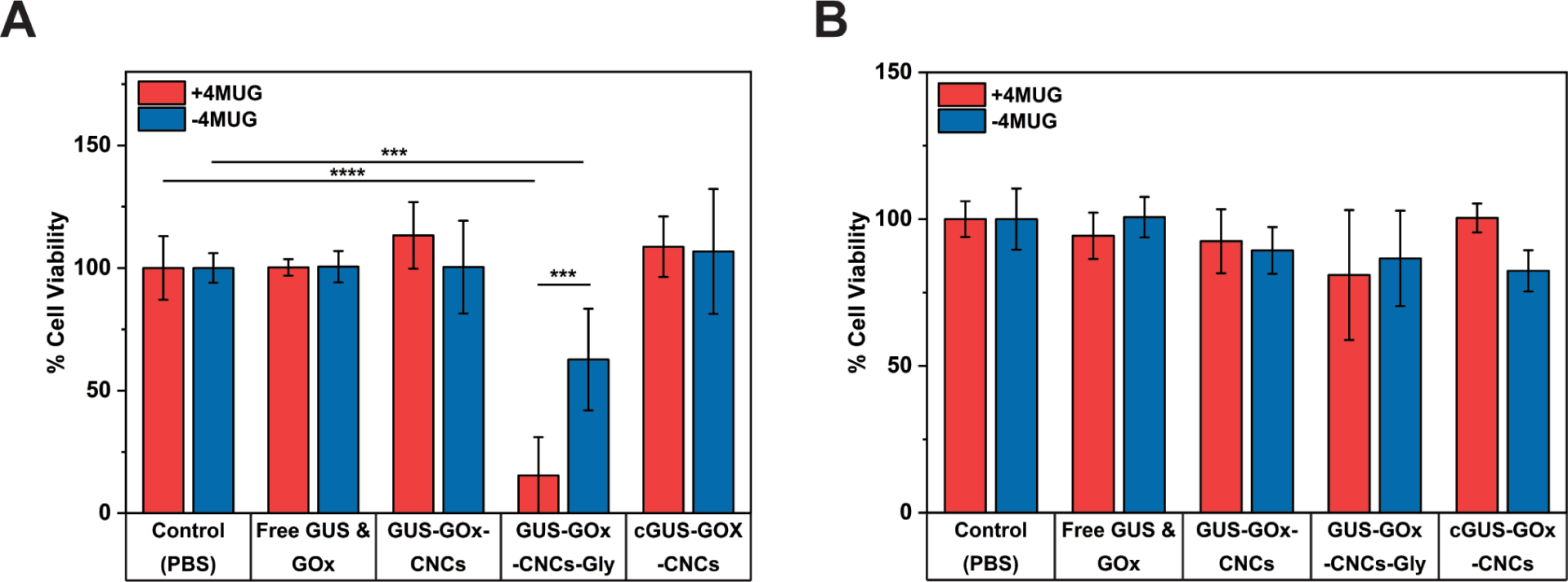
Cell viability as percentage of (A) HepG2 and (B) HeLa S3 H2B-GFP cells. Graph shows mean ± s.d. of 3 experiments.

The synergistic potential of hymecromone in combination with sorafenib has been well investigated, revealing effects such as decreasing cell proliferation and motility, and inducing apoptosis and capillary formation in tumors.^87^ However, the synergy of hymecromone with H_2_O_2_ has not yet been explored. To investigate how the simultaneous production of intracellular hymecromone and H_2_O_2_ by our CNCs influences cell viability, MTS cell proliferation assays were performed (Figure 8). First, the incubation effects of the CNCs, empty polymersomes, glycooligomer and enzymes on cell viability after 24 h were examined (Figure S26). GUS-GOx-CNCs, empty polymersomes, glycooligomer, and free GUS or GOx were not cytotoxic as displayed by the lack of negative impact on the viability of HepG2 and HeLa S3 H2B-GFP cells. Subsequently, we investigated the synergistic effect of hymecromone and H_2_O_2_ production on cell viability. To note, glucose was constant in our experiments, as it is a necessary component of the cell culture medium and supports the mimicking of the glucose-rich tumor microenvironment.^88^ To investigate the potential synergy, we controlled the providing of 4-MUG. No significant decrease in cell viability was observed for the cells incubated with GUS-GOx-CNCs and nonpermeabilized CNCs regardless of 4-MUG addition, indicating limited cellular uptake, lack of intracellular hymecromone production and significantly lower ROS levels (Figures 5, 7 and 8A). Similarly, cells incubated with free GUS/GOx presented no decreased viability, associated with low intracellular ROS and hymecromone amounts (Figures 7 and 8A). The highest decrease (85% ± 15%) was observed in HepG2 cells incubated with GUS-GOx-CNCs-Gly and 4-MUG (Figure 8A). Importantly, cells incubated with GUS-GOx-CNCs-Gly but without 4-MUG addition, showed 37% ± 21% reduction in cell viability which is attributed to the intracellularly produced H_2_O_2_ (Figure 8A). It is noteworthy that cells incubated with 190 μM hymecromone, experienced only 27% ± 3% decrease in viability, which is in accordance to previous studies investigating the antitumoral effects of hymecromone (Figure S27).^81,89^ In combination, these results highlight two main aspects of our glycooligomer-functionalized nanosystem: the crucial role of the glycooligomer unit in enhancing cellular uptake in HepG2 cells and the subsequent synergistic effect of the bioorthogonally generated hymecromone and H_2_O_2_ on cell viability. In contrast, HeLa S3 H2B-GFP cells exhibited no significant decrease in viability when incubated with differently loaded nanocompartments or free enzymes, regardless of 4-MUG addition (Figure 8B). The low cytotoxicity in HeLa S3 H2B-GFP is in agreement with the cell uptake assays and intracellular hymecromone/H_2_O_2_ production in this cell line. These results further underline the importance of the glycooligomer unit in the efficient, cell-specific uptake and the resulting cytotoxic effects of our catalytic nanocompartments.

## Conclusions

Our study introduces for the first time multifunctional catalytic nanocompartments to produce a synergistic effect with controlled localization at the target site. The catalytic nanocompartments efficiently catalyze two independent reactions in parallel through coencapsulation of two enzymes, β-glucuronidase and glucose oxidase, which simultaneously produce the cytotoxic drug hymecromone from its glucuronide conjugate and H_2_O_2_ through glucose consumption, respectively. The selective cell internalization in HepG2 is driven by the glycooligomer functionalization of the compartments, serving the interaction with the overexpressed mannose-binding receptors. The synergistic effect of hymecromone and H_2_O_2_ resulted in a significant reduction of HepG2 cell viability. Together, targeted and multifunctional compartments combining bioorthogonal reactions – such as drug production and cell starvation – provide optimal response and inspire future strategies and applications in the fast-developing field of drug synergism. The promise of our nanosystem lies in its potential to refine and steer the direction in enzyme-based therapeutic approaches, by the targeted activity of two coencapsulated enzymes that work in parallel and on-demand for the production of the desired compounds, leading to an improved therapeutic outcome. This study sets the stage for further exploration and development of such nanosystems, contributing to the ongoing demand for innovative and combinatorial strategies in cancer therapy.

## Supporting Information Available

The NMR spectra of the diblock copolymers, synthesis procedure, and characterization of the glycooligomer, additional physiochemical characterization data of the nanocompartments, calibration curves, SPR binding curves, additional enzymatic assays, stability data, additional cell CLSM micrographs and analysis, and additional cell viability assays (PDF)
