# Supplementary material for: Glycooligomer-Functionalized Catalytic Nanocompartments Co-Loaded with Enzymes Support Parallel Reactions and Promote Cell Internalization
Source: Biomacromolecules. 2024 Jun 24;25(7):4492–509. doi: 10.1021/acs.biomac.4c00526 (PMC11238334; doi:10.1021/acs.biomac.4c00526)
Supplement: Supplementary file 1 — bm4c00526_si_001.pdf [file bm4c00526_si_001.pdf]

Supporting Information

Glycooligomer-functionalized Catalytic Nanocompartments Co-loaded with Enzymes Support Parallel Reactions and Promote Cell Internalization

Maria Korpidou,[a] Jonas Becker,[b] Shabnam Tarvirdipour,[a] Ionel Adrian Dinu,[a] C. Remzi Becer[b] and Cornelia G. Palivan[ac]**

[a] Department of Chemistry, University of Basel, Mattenstrasse 22, Basel, 4002, Switzerland

[b] Department of Chemistry, University of Warwick, Coventry, CV4 7AL, United Kingdom

[c] NCCR Molecular Systems Engineering, Mattenstrasse 22, Basel, 4002, Switzerland

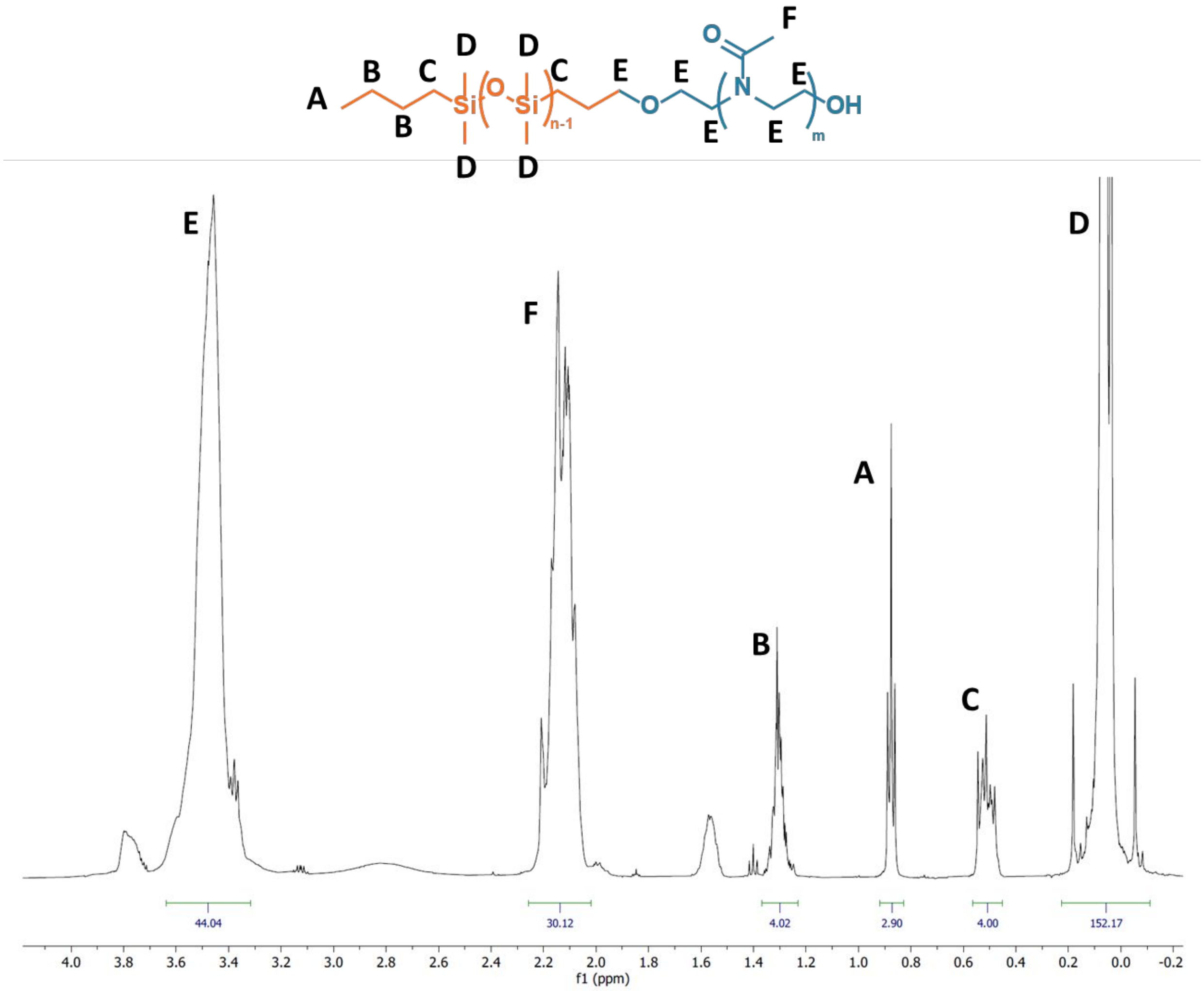

Figure S1. ^{1}H NMR spectrum of PDMS$_{25}$-*b*-PMOXA$_{10}$-OH (500 MHz, MeOD, 295 K, δ, ppm).

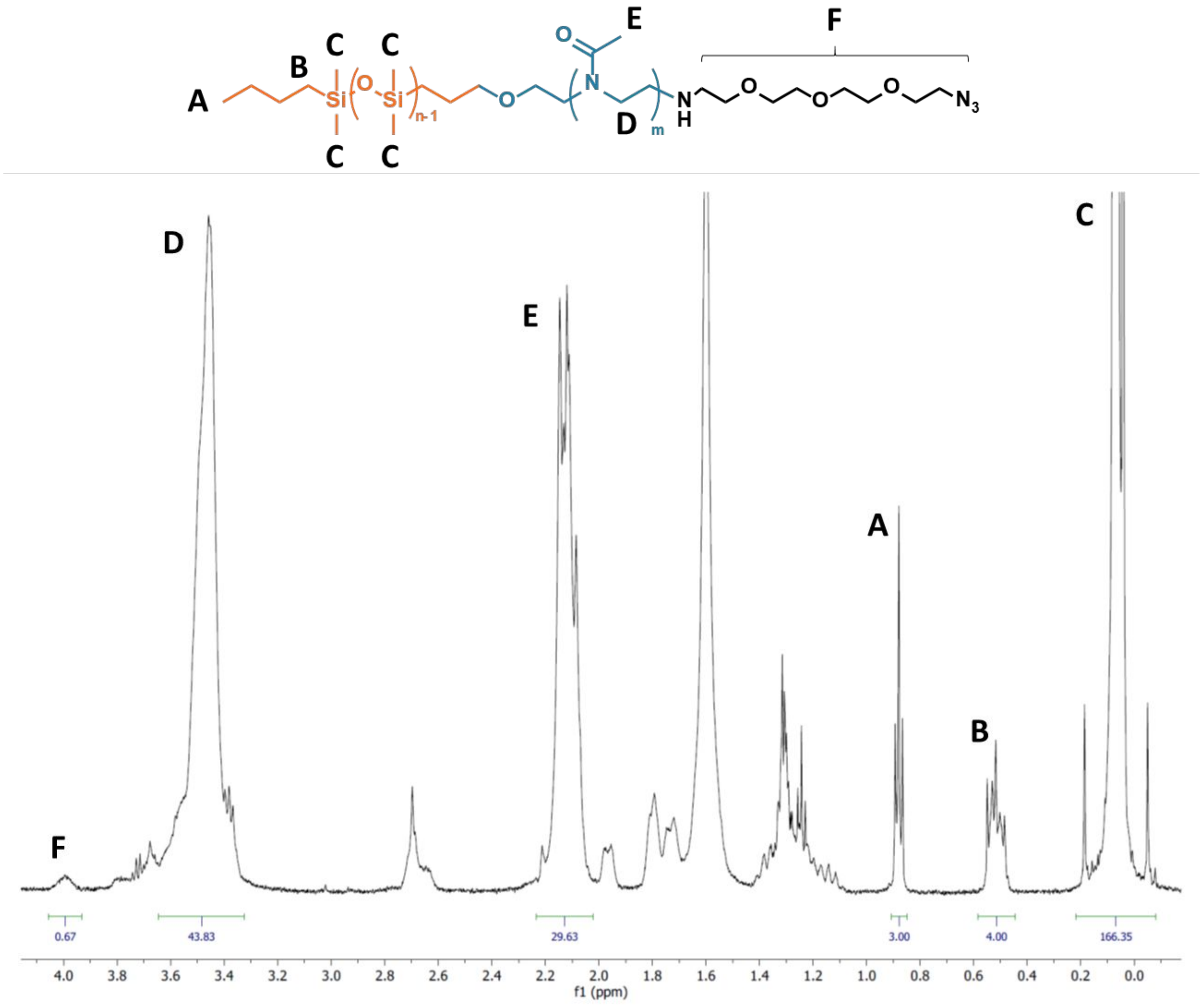

Figure S2. ^{1}H NMR spectrum of $PDMS_{22}$-*b*-$PMOXA_8$-N_3 (500 MHz, $CDCl_3$, 295 K, δ, ppm).

Synthesis of glycooligomer with eight mannose units

Synthesis of **2**, acetylated glycooligomer:

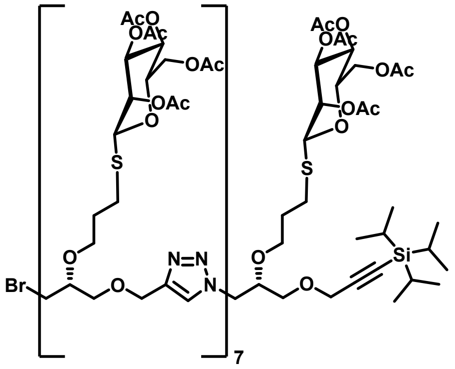

Under an inert gas atmosphere, **1** (0.200 g, 0.114 mmol, 1.0 equiv.), 2,2-Dimethoxy-2-phenylacetophenone (0.234 g, 0.91 mmol, 8.0 equiv.) and Ac_4ManSH (1.994 g, 5.47 mmol, 48.0 equiv., 6.0 eq per ene) were suspended in anhydrous acetonitrile (3.0 mL) and degassed by flushing with N_2 for 5 min. The mixture was irradiated with UV light (365 nm) for 18 h. The solvent was removed under reduced pressure and the crude product was purified by silica gel column chromatography (pure EtOAc to DCM/MeOH 5%) and **2** was obtained as a white solid (0.430 g, 81%).

^{1}H NMR (400 MHz, $CDCl_3$): δ(ppm) = 7.66 (s, 7H), 5.52-5.44 (m, 8H), 5.31-5.19 (11H), 5.14-5.04 (m, 8H), 4.84-4.77 (m, 7H), 4.71-4.51 (m, 24H), 4.48-4.34 (m, 8H), 4.26 (dd, J = 12.2, 5.4 Hz, 9H), 4.18-4.04 (m, 13H), 3.93-3.80 (bs, 8H), 3.80-3.68 (bs, 10H), 3.68-3.48 (m, 27H), 3.47-3.32 (bs, 10H), 2.66 (t, J = 6.5 Hz, 12H), 2.17 (s, 24H), 2.06 (s, 24H), 2.04 (s, 24H), 1.96 (s, 24H), 1.86-1.71 (m, 18H), 1.09-1.00 (m, 21H).

Synthesis of **3**, deacetylated glycooligomer:

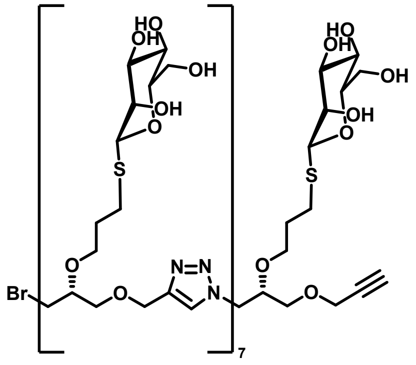

2 (0.304 g, 0.065 mmol) was dissolved in anhydrous THF (1 mL) and Tetrabutylammonium fluoride solution (1 M in THF, 0.065 mL, 1.0 equiv.) was added. The mixture was stirred at room

temperature for 4 h. DCM (50 mL) was added and washed with H_2O (3 x 50 mL). The organic phase was dried over $MgSO_4$, and the solvent removed under reduced pressure. The intermediate product was dissolved in MeOH (10 mL) and sodium methoxide solution (25 wt.% in MeOH, 0.2 mL) was added. The mixture was stirred for 16 h, then MeOH was removed under reduced pressure and the crude product was dissolved in H_2O (10 mL). It was transferred into a dialysis membrane (MWCO 1kDa) and dialyzed against H_2O for 2d and lyophilized. Final product **3** was obtained as a white solid (0.048 g, 23%).

^{1}H NMR (400 MHz, $CDCl_3$): δ(ppm) = 8.07 (s, 7H), 4.73-4.45 (m, 29H), 4.26 (bs, 1H), 4.11-3.94 (m, 12H), 3.93-3.83 (m, 8H), 3.81-3.52 (m, 42H), 3.50-3.31 (m, 12H), 2.94 (bs, 1H), 2.81 (bs, 1H), 2.65-2.46 (m, 8H), 1.74 (bs, 12H), 1.31 (bs, 20H), 0.91 (bs, 16H)

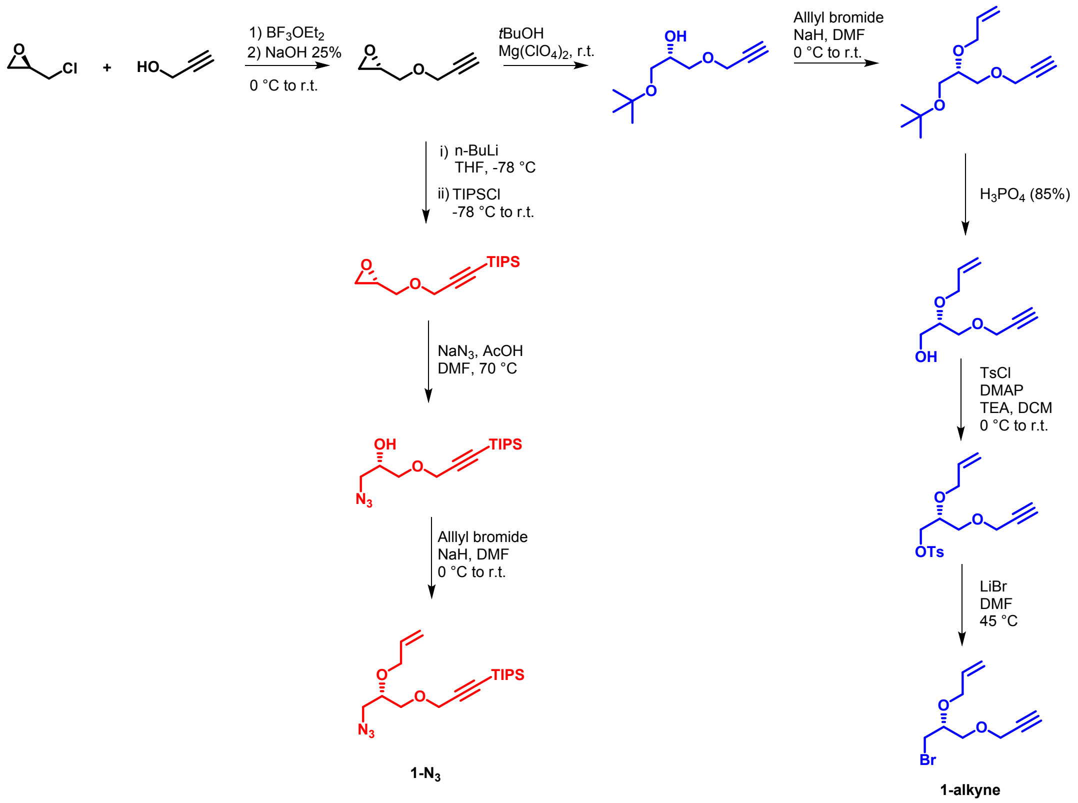

Figure S3. Reaction scheme for the synthesis of IEG precursors developed by Johnson *et al.* [1–4]

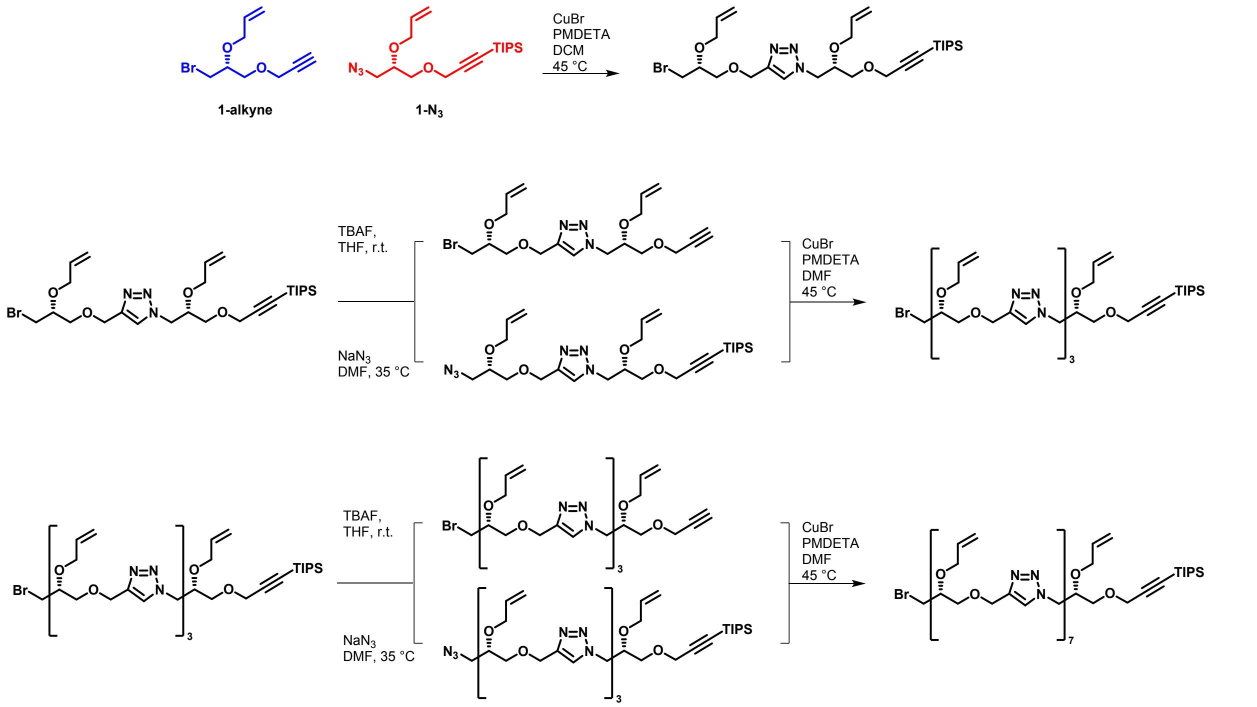

Figure S4. Reaction scheme of the Iterative Exponential Growth (IEG) of allyl-IEGmers developed by Johnson *et al.* [1–4]

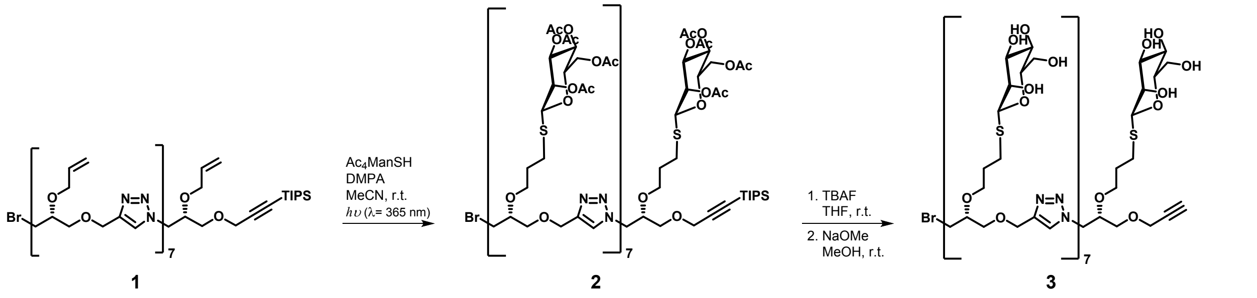

Figure S5. Reaction scheme of the functionalization of allyl-IEG octamer with thiomannose (Ac_4ManSH) and subsequent deprotection.

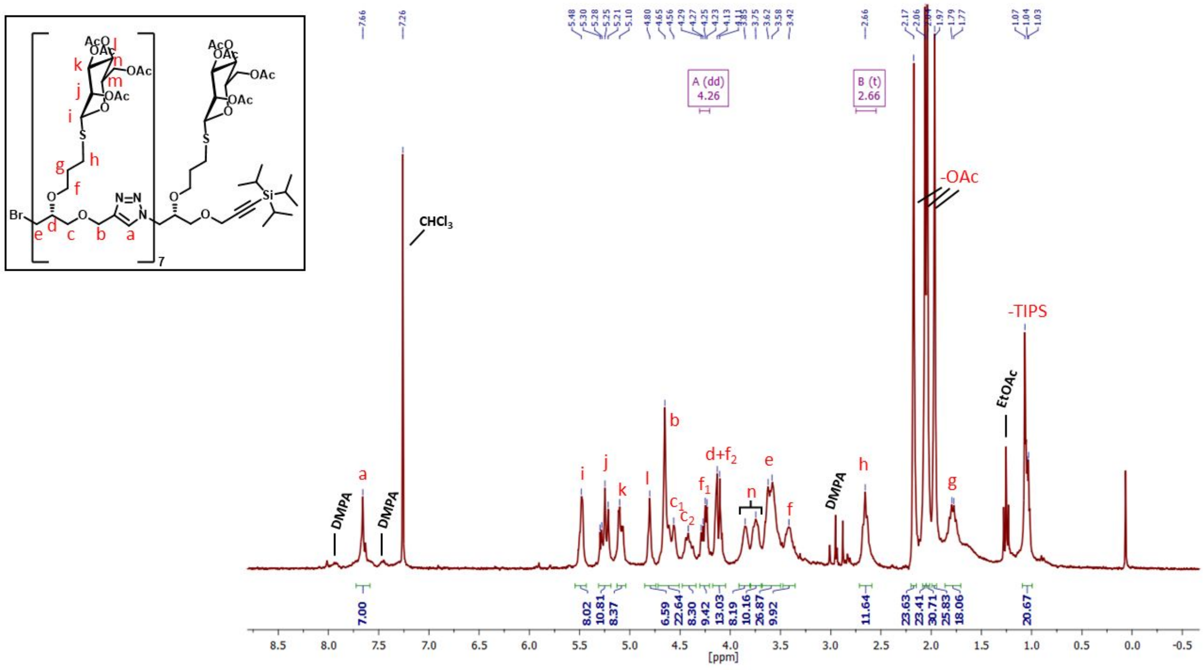

Figure S6. ^{1}H NMR spectrum of **2** in $CDCl_3$.

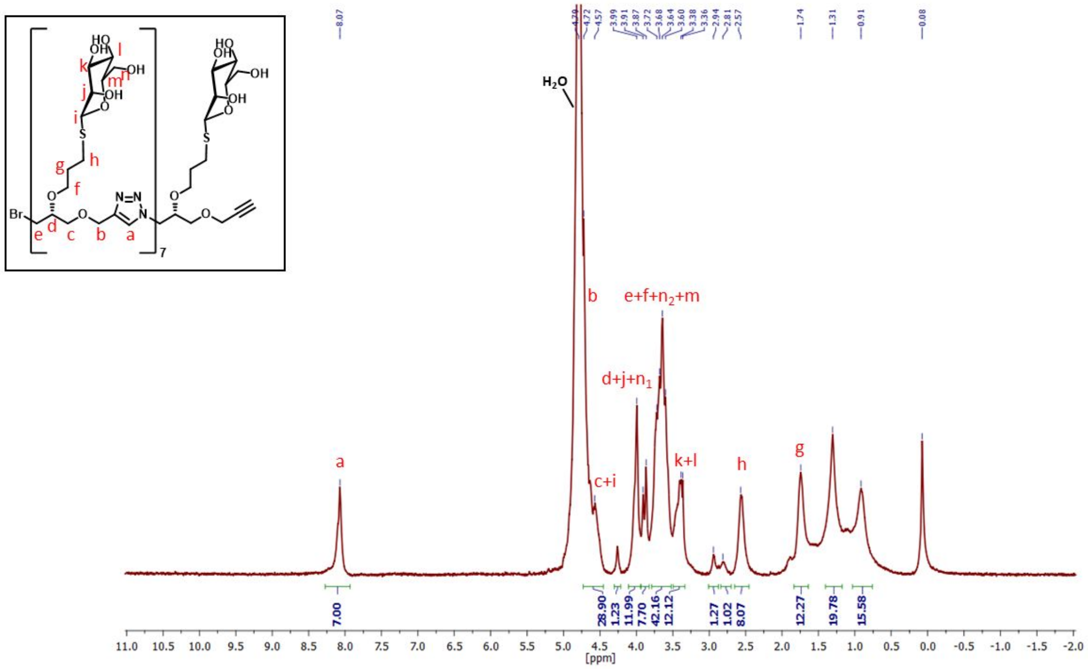

Figure S7. ^{1}H NMR spectrum of **3** in D_2O.

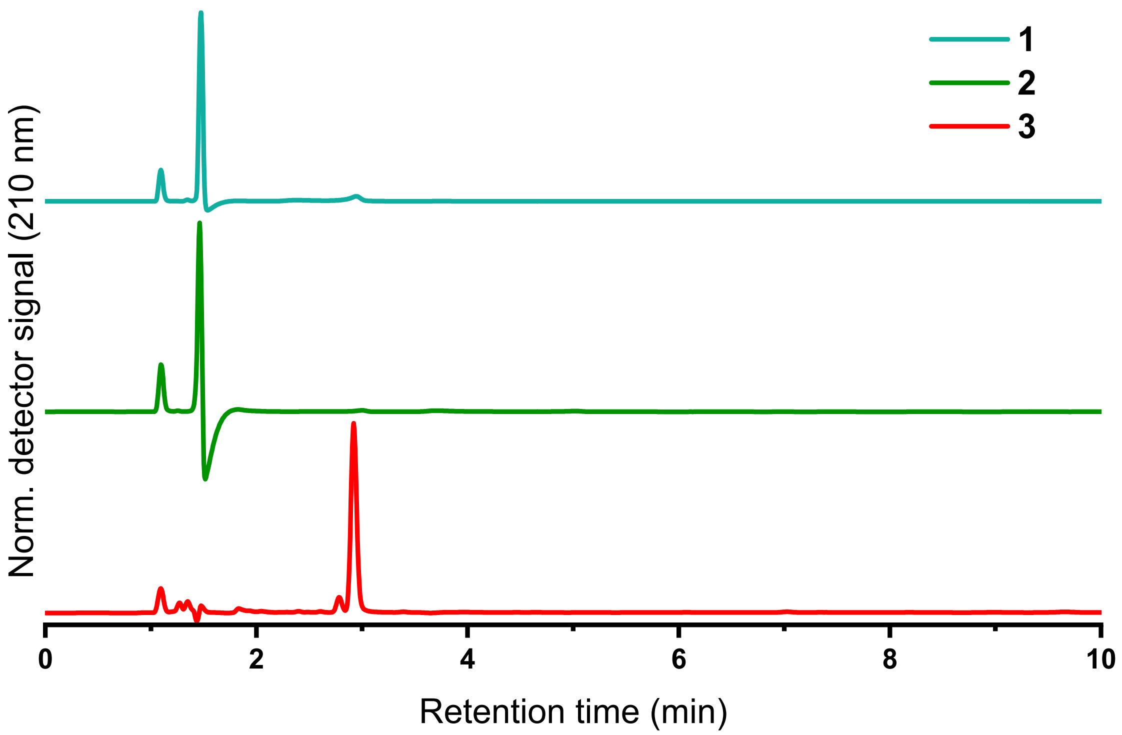

Figure S8. HPLC analysis (method: A/B = 99/1, A: H_2O with 0.04% TFA, B: MeCN with 0.04% TFA) of allyl-octamer **1**, glycooligomer **2** and deprotected glycooligomer **3**.

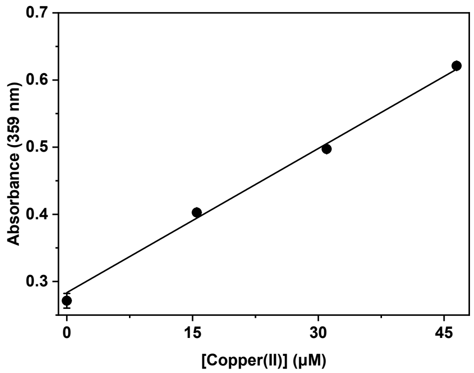

Figure S9. Copper(II) calibration curve ($R^2 = 0.99$).

Table S1. Characterization of polymersomes

	Diameter (nm) by DLS	PDI	Diameter (nm) by NTA	ρ (R_g/ R_h)	Concentration (polymersomes mL^{-1})
**Glycooligomer-functionalized polymersomes**	135 ± 54	0.1	138 ± 38	0.81 ± 0.16	(5.6 ± 0.5) x 10^{11}
**Non-functionalized polymersomes**	122 ± 40	0.07	108 ± 22	0.97 ± 0.05	(7.9 ± 0.5) x 10^{11}
**Glycooligomer-functionalized Atto647-encapsulating polymersomes**	137 ± 39	0.05	140 ± 26	1.05 ± 0.23	(6.7 ± 0.1) x 10^{11}
**Non-functionalized Atto647-encapsulating polymersomes**	128 ± 40	0.1	112 ± 23	1.03 ± 0.1	(6.5 ± 0.18) x 10^{11}

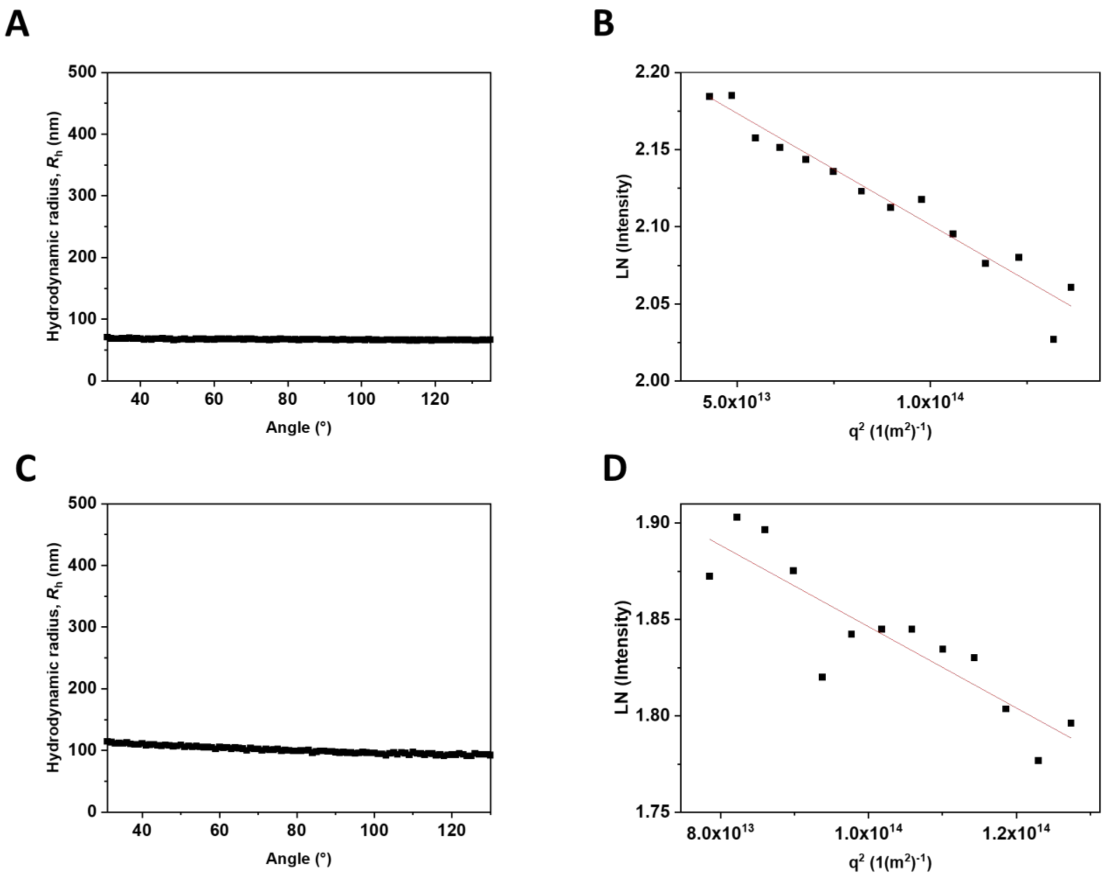

Figure S10. DLS profile of (A) non-functionalized and (C) glycooligomer-functionalized polymersomes showing the mean hydrodynamic radius, R_h, SLS data of (B) non-functionalized and (D) glycooligomer-functionalized polymersomes and linear fit to the Guinier equation.

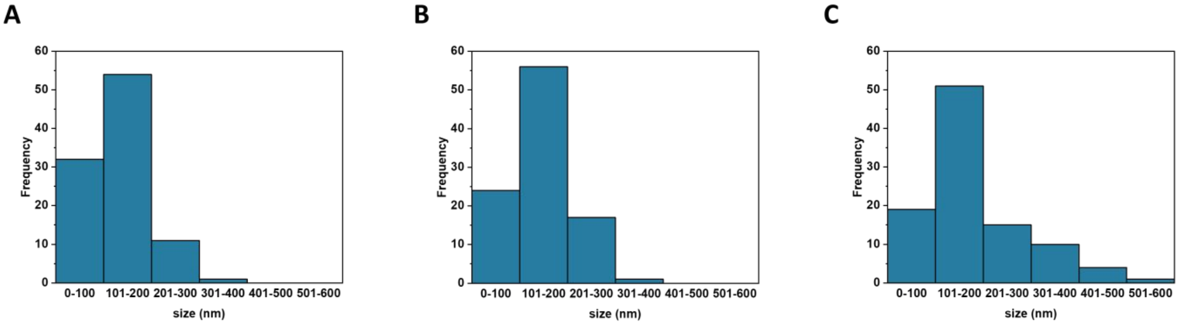

Figure S11. Histogram showing the size distribution (diameter) of (A) non-fnctionalized polymersomes, (B) glycooligomer-functionalized polymersomes and (C) GUS-GOx-CNCs-Gly. The data were obtained by measuring the diameter of 100 nanocompartments for each set in TEM micrographs.

Table S2. Number of melittin pores per polymersome.

Sample	Diffusion time (µs)	No. of melittin pores/ polymersome
PSs-Cy5-mel (25 µM)	4754 ± 1712	133 ± 12
PSs-Cy5-mel (50 µM)	4289 ± 1501	242 ± 28
PSs-Cy5-mel (75 µM)	5533 ± 2170	225 ± 28
PSs-Cy5-mel-Gly (50 µM)	4959 ± 1975	220 ± 16

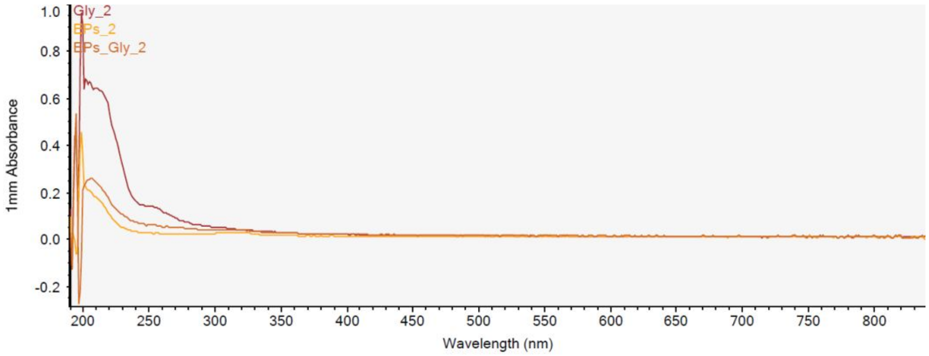

Figure S12. Representative UV-Vis spectra of glycooligomer **3** (Gly), empty, non-functionalized polymersomes (EPs) and glycooligomer-functionalized polymersomes (EPs_Gly).

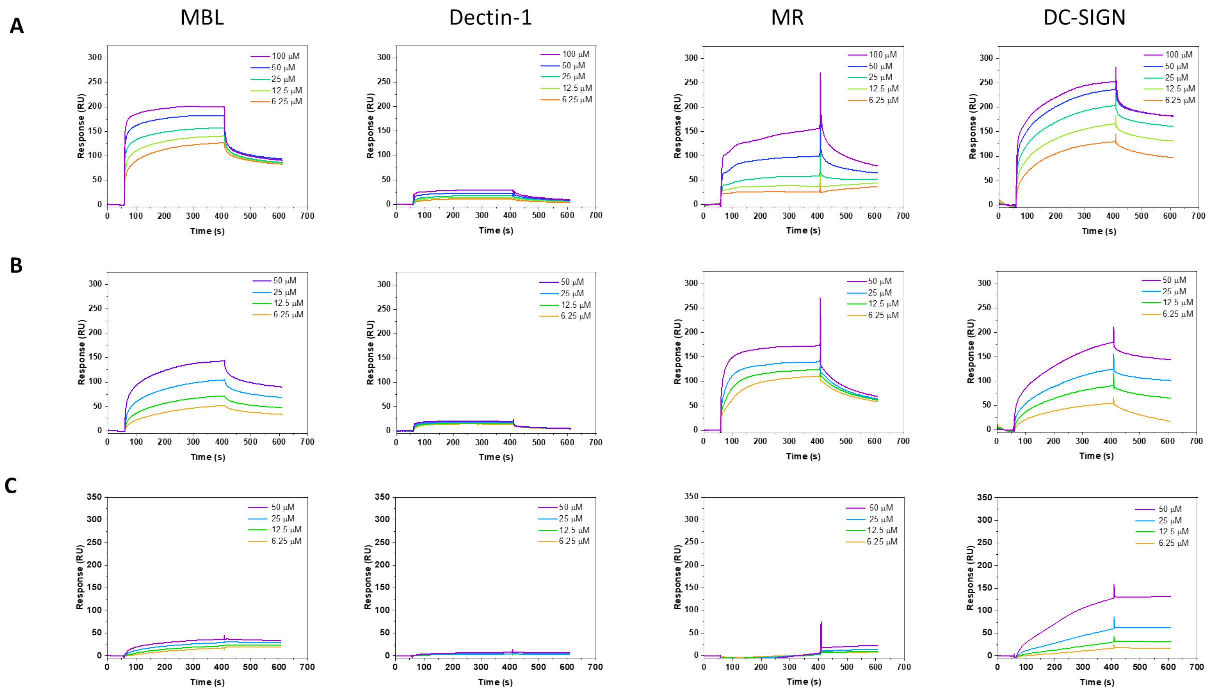

Figure S13. Binding curves obtained by Surface Plasmon Resonance (SPR) analysis of (A) glycooligomer, (B) glycooligomer-functionalized polymersomes and (C) non-functionalized polymersomes.

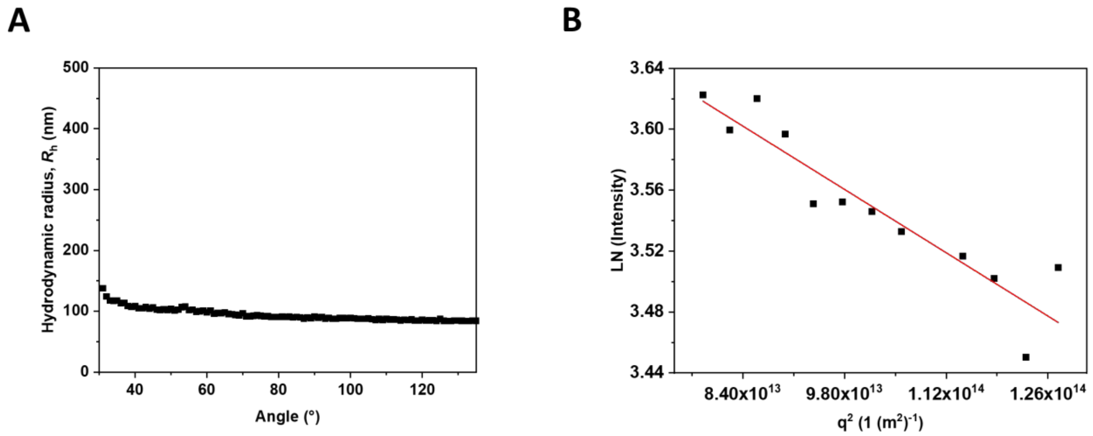

Figure S14. A. DLS profile of GUS-GOx-CNCs-Gly showing the mean hydrodynamic radius, R_h, B. SLS data of GUS-GOx-CNCs-Gly and linear fit to the Guinier equation.

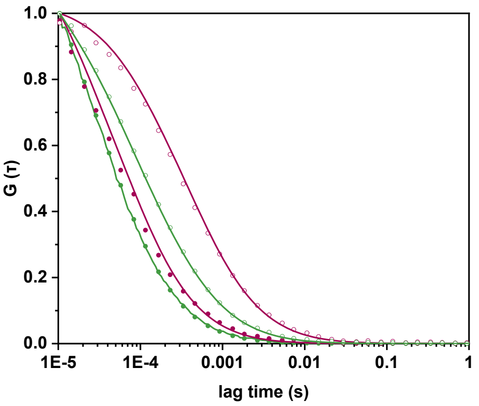

Figure S15. Normalized FCS autocorrelation curves for free Atto488 (green, full symbols), free Atto633 (red, full symbols), GUS-Atto488 (green, empty symbols) and GOx-Atto633 (red, empty symbols). Symbols: raw data, Solid lines: fitted curves.

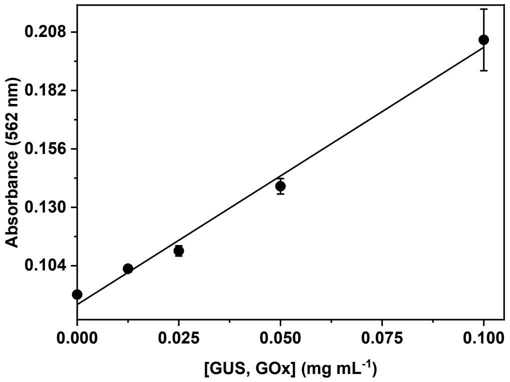

Figure S16. GUS, GOx calibration curve for the BCA protein assay ($R^2 = 0.99$).

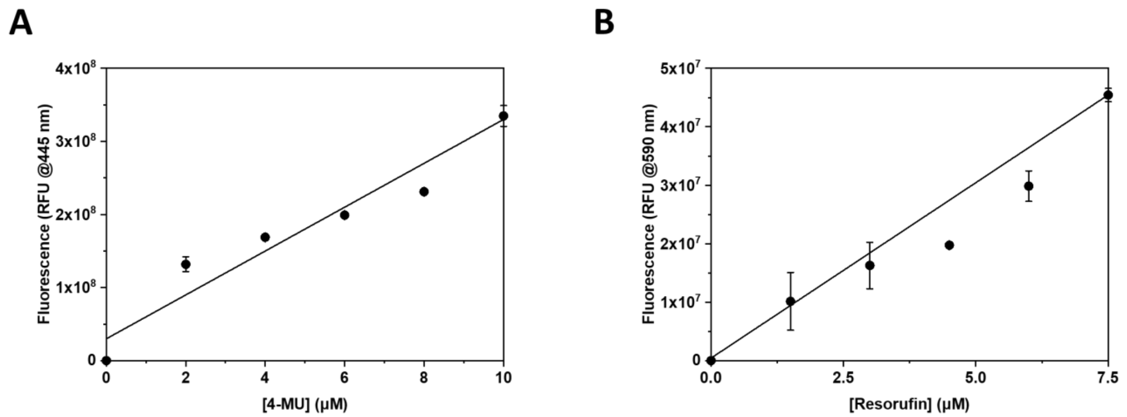

Figure S17. Calibration curves using (A) 4-MU ($R^2 = 0.93$) and (B) resorufin standards ($R^2 = 0.96$) in PBS containing 50% Dulbecco's Modified Eagle Medium (DMEM) Phenol Red free with 10% Fetal Bovine Serum.

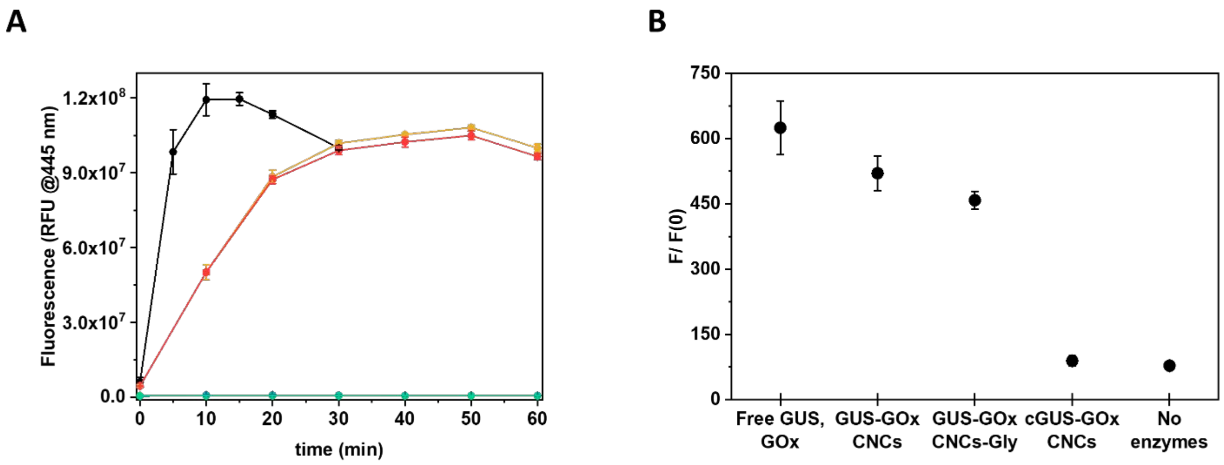

Figure S18. Enzymatic efficiency of CNCs in PBS at 37 °C A. 4-MUG (10 μM) conversion to 4-MU, black: free enzymes, yellow: GUS-GOx-CNCs, red: GUS-GOx-CNCs-Gly, green: non-permeabilized

CNCs, blue: reaction mix without enzymes, B. Amplex™ Red (10 µM) conversion to resorufin. Mean ± s.d. of 3 measurements.

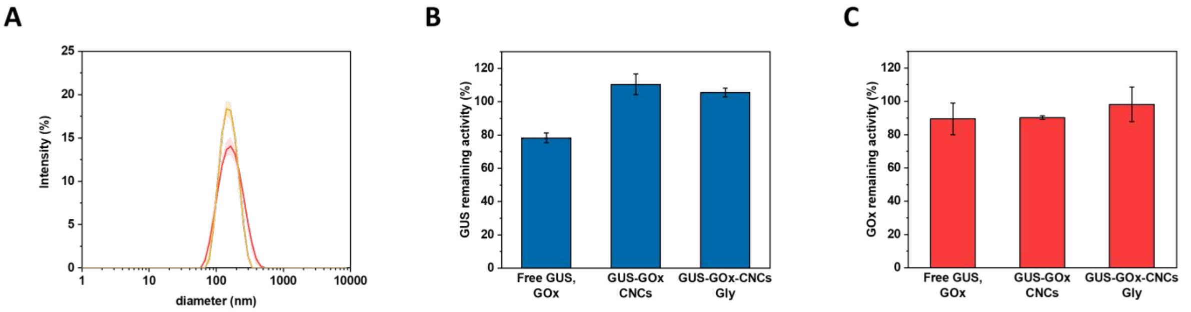

Figure S19. Size distribution of (A) GUS-GOx-CNCs (yellow) and GUS-GOx-CNCs-Gly (red) measured by DLS, after storage for 2 months at 4 °C. Remaining percentages of activity of (B) GUS and (C) GOx upon storage at 4 °C for 2 months, Measurements represent the mean ± s.d.

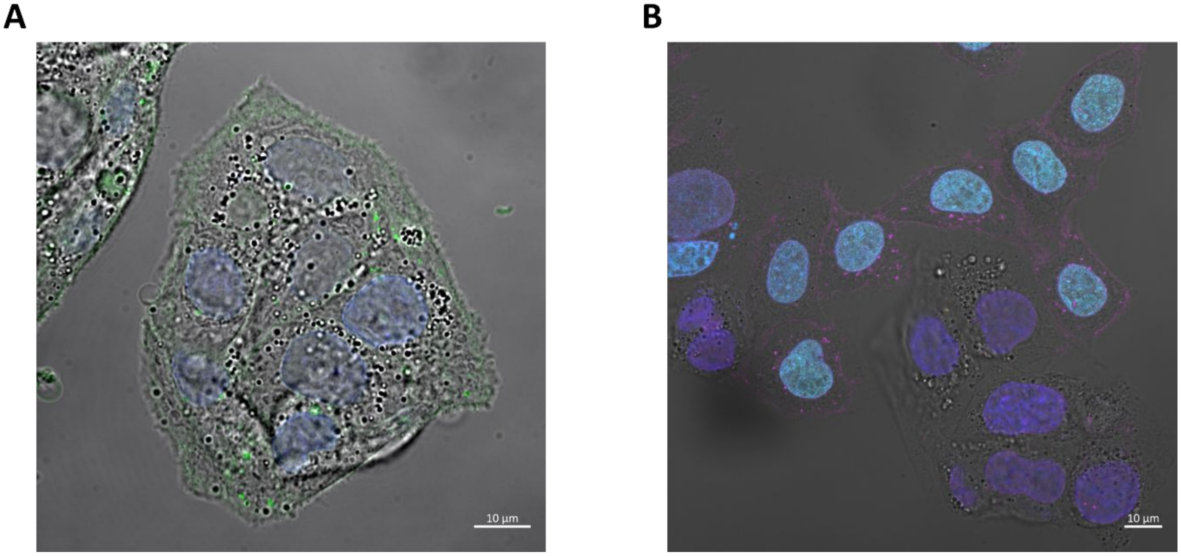

Figure S20. A. HepG2 cells incubated with the equivalent amount of PBS, Yellow: polymersomes, Atto647, Green: Cell membranes, Atto488-WGA, Blue: nuclei, Hoechst 33342, B. HepG2 and HeLa S3 H2B-GFP co-cultured cells incubated with the equivalent amount of PBS, Yellow: polymersomes, Atto647, Pink: Cell membranes, Atto555-WGA, Blue: nuclei, Hoechst 33342, Green: nuclei, GFP. Scale bar: 10 µm.

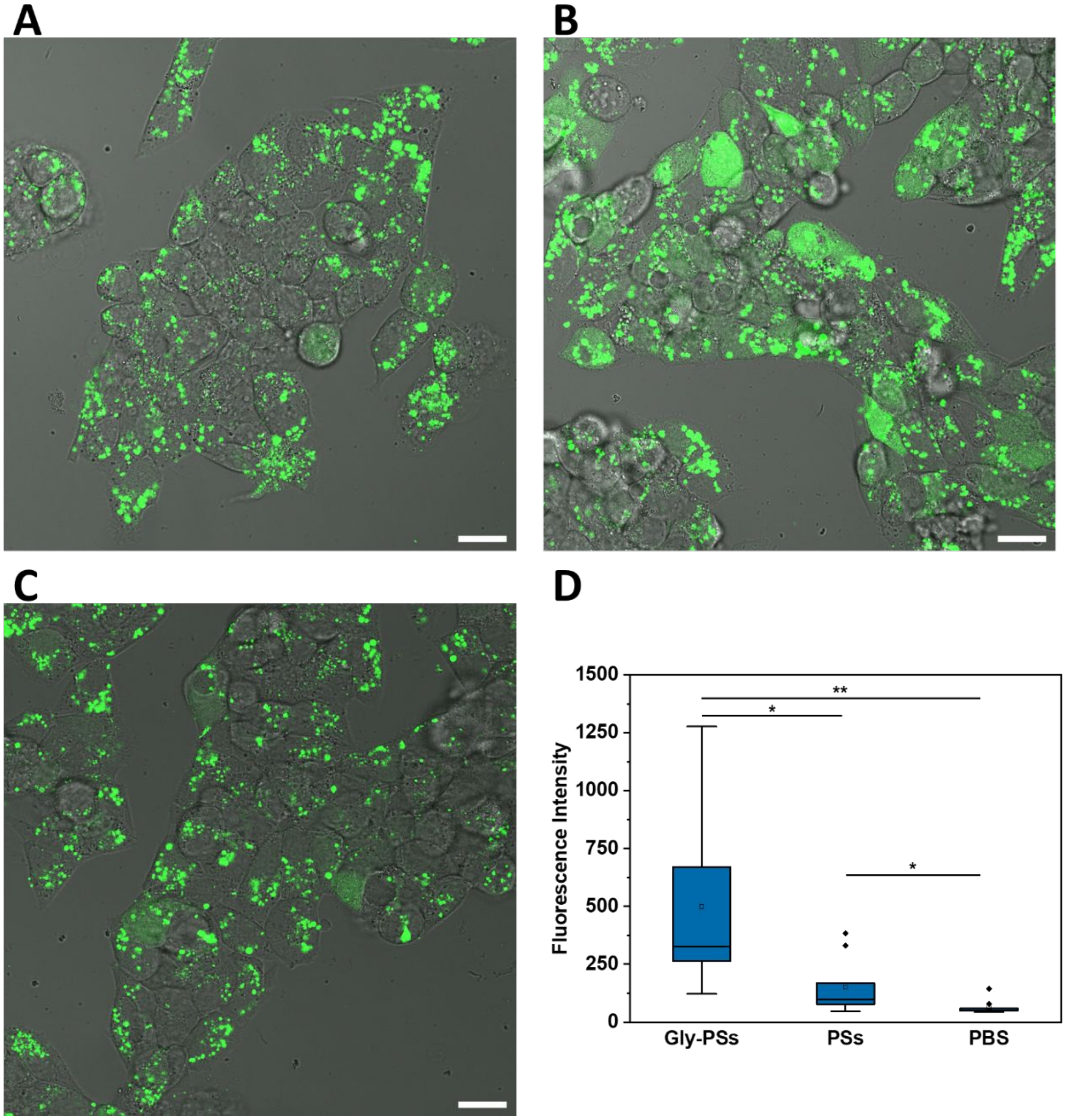

Figure S21. Endosomal escape of polymersomes in HepG2 cells. A. PBS-incubated cells, B. glycooligomer-functionalized polymersomes-incubated cells, C. non-functionalized polymersomes-incubated cells for 4 hours with Calcein (250 μM), D. Fluorescence intensity of calcein as measured in the cytoplasm of HepG2 cells by CLSM micrographs (Gly-PSs: glycooligomer-functionalized polymersomes, PSs: non-functionalized polymersomes). Green: Calcein. Scale bar: 20 μm.

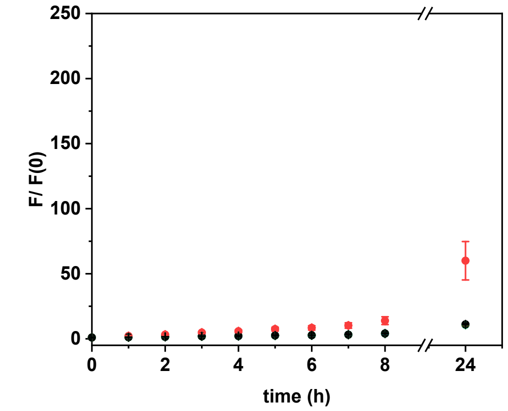

Figure S22. Catalytic nanocompartments in HeLa S3 H2B-GFP cells. A. Intracellular production of hymecromone after incubation with GUS-GOx-CNCs (yellow), GUS-GOx-CNCs-Gly (red), cGUS-GOx-CNCs (green), free GUS/ GOx mixture (blue) or PBS (black) for 24h. Cells were washed and exposed to a single dose of 500 µM 4-MUG. Fluorescence was recorded for 24 hours. Graph shows mean ± s.d. of three experiments.

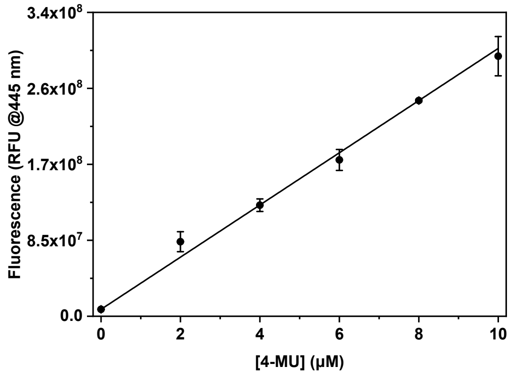

Figure S23. Calibration curves using 4-MU in full Dulbecco's Modified Eagle Medium, $R^2 = 0.99$.

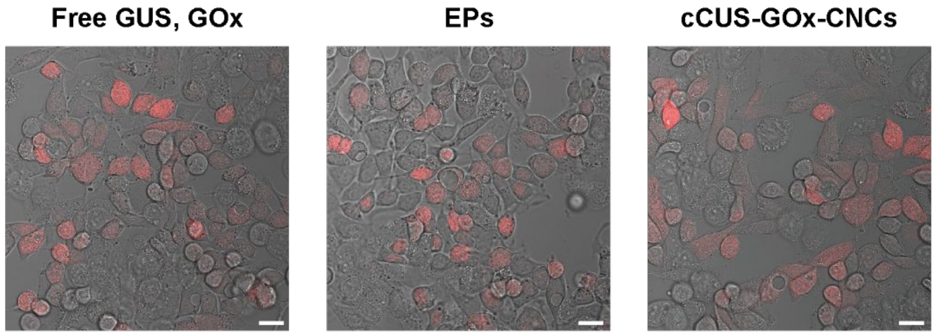

Figure S24. CLSM micrographs of 2’,7’-dichlorodihydrofluorescein diacetate incubated HepG2 cells. Red: ROS species, DCF. Scale bar: 20 μm.

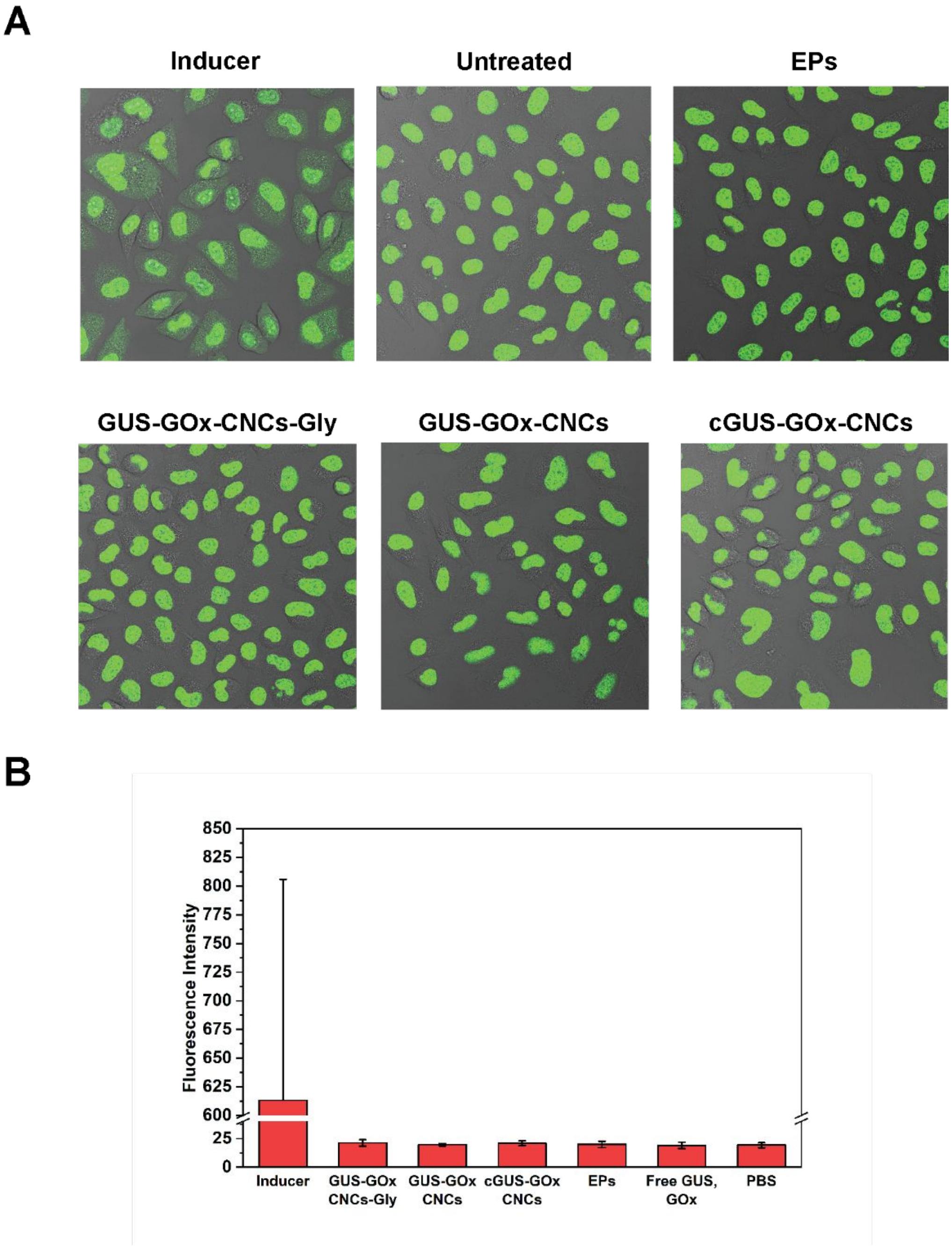

Figure S25. A. CLSM micrographs of 2’,7’-dichlorodihydrofluorescein diacetate incubated HeLa S3 H2B-GFP cells. Green in cytoplasm: ROS species, DCF. Scale bar: 20 μm. B. Total fluorescence intensity of DCF as analyzed by CLSM micrographs. Graph shows mean ± s.d. of three micrographs.

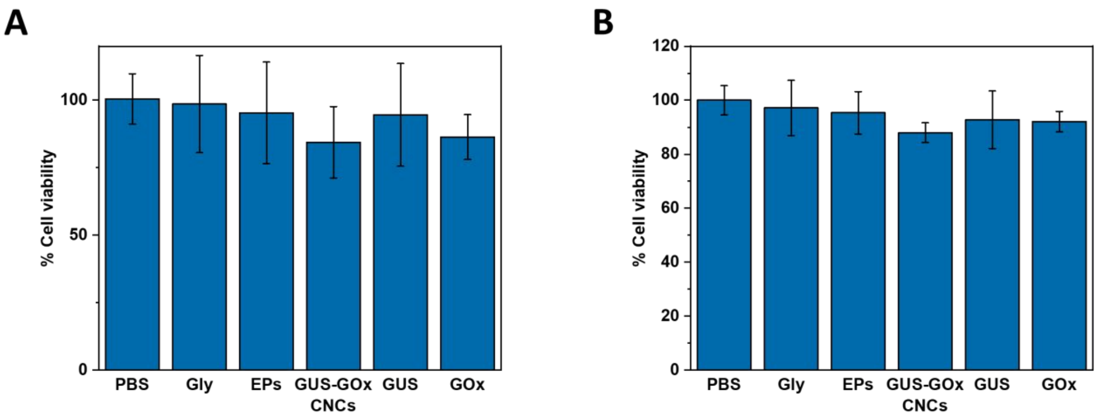

Figure S26. Cell viability as percentage of (A) HepG2 and (B) HeLa S3 H2B-GFP cells incubated with only PBS (control), glycooligomer, empty polymersomes, GUS-GOx-CNCs, GUS or GOx. Graph shows mean ± s.d. of six repetitions.

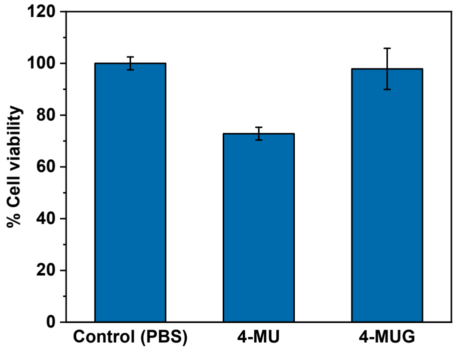

Figure S27. Cell viability as percentage of HepG2 cells incubated with only PBS (control), 4-MU (190 μM) or 4-MUG (190 μM). Graph shows mean ± s.d. of six repetitions.

References:

(1) Barnes, J. C.; Ehrlich, D. J. C.; Gao, A. X.; Leibfarth, F. A.; Jiang, Y.; Zhou, E.; Jamison, T. F.; Johnson, J. A. Iterative Exponential Growth of Stereo- and Sequence-Controlled Polymers. *Nat. Chem.* **2015**, *7* (10), 810–815.

(2) Jiang, Y.; Golder, M. R.; Nguyen, H. V. T.; Wang, Y.; Zhong, M.; Barnes, J. C.; Ehrlich, D. J. C.; Johnson, J. A. Iterative Exponential Growth Synthesis and Assembly of Uniform Diblock Copolymers. *J. Am. Chem. Soc.* **2016**, *138* (30), 9369–9372.

(3) Golder, M. R.; Jiang, Y.; Teichen, P. E.; Nguyen, H. V. T.; Wang, W.; Milos, N.; Freedman, S. A.; Willard, A. P.; Johnson, J. A. Stereochemical Sequence Dictates Unimolecular Diblock Copolymer Assembly. *J. Am. Chem. Soc.* **2018**, *140* (5), 1596–1599.

(4) Hartweg, M.; Jiang, Y.; Yilmaz, G.; Jarvis, C. M.; Nguyen, H. V. T.; Primo, G. A.; Monaco, A.; Beyer, V. P.; Chen, K. K.; Mohapatra, S.; Axelrod, S.; Gómez-Bombarelli, R.; Kiessling, L. L.; Becer, C. R.; Johnson, J. A. Synthetic Glycomacromolecules of Defined Valency, Absolute Configuration, and Topology Distinguish between Human Lectins. *JACS Au* **2021**, *1* (10), 1621–1630.
